# Serum fatty acid profiles in breast cancer patients following treatment

**DOI:** 10.1186/s12885-023-10914-2

**Published:** 2023-05-12

**Authors:** Alicja Pakiet, Agata Jędrzejewska, Katarzyna Duzowska, Alina Wacławska, Patrycja Jabłońska, Jacek Zieliński, Adriana Mika, Tomasz Śledziński, Ewa Słomińska

**Affiliations:** 1 Department of Environmental Analysis, Faculty of Chemistry, University of Gdańsk, Wita Stwosza 63, 80-308 Gdańsk, Poland; 2 Department of Biochemistry, Medical University of Gdańsk, Dębinki 1, 80-211 Gdańsk, Poland; 3 Department of Pharmaceutical Biochemistry, Medical University of Gdańsk, Dębinki 1, 80-211 Gdansk, Poland; 4 Department of Surgical Oncology, Medical University of Gdansk, Mariana Smoluchowskiego 17, 80-214 Gdańsk, Poland

**Keywords:** Breast cancer, Fatty acids, Lipids, Serum, Gas chromatography-mass spectrometry

## Abstract

**Background:**

Breast cancer is associated with alterations in lipid metabolism. The treatment of breast cancer can also affect serum lipid composition. The purpose of this study was the examination of serum fatty acids (FAs) profiles in breast cancer survivors to assess if the FA levels normalize.

**Methods:**

Serum levels of FAs were determined by gas chromatography–mass spectrometry in a group of breast cancer patients at baseline (before treatment, *n* = 28), at two follow-up visits at 12 months (*n* = 27) and 24 months (*n* = 19) after the breast cancer resection, and in the group of healthy controls (*n* = 25). Multivariate analysis was performed to assess how FA serum profile changes following treatment.

**Results:**

Breast cancer patients’ serum FA profiles at follow-ups did not normalize to the levels of control group. The greatest differences were found for levels of branched-chain (BCFA), odd-chain (OCFA) and polyunsaturated (PUFAs) FAs, all of which were significantly increased 12 months after the surgery.

**Conclusions:**

After treatment for breast cancer, the patients’ serum FA profile differs from the profile before treatment and from controls, especially 12 months after treatment. Some changes may be beneficial – increased BCFA and OCFA levels, and improved n-6/n-3 PUFA ratio. This may reflect lifestyle changes in breast cancer survivors and have an impact on the risk of recurrence.

**Supplementary Information:**

The online version contains supplementary material available at 10.1186/s12885-023-10914-2.

## Background

The female breast cancer (BC) is the most commonly diagnosed type of cancer worldwide [1]. The high incidence coupled with increased use of diagnostic screening mammography and improvement in breast cancer treatment resulted in increased numbers of breast cancer survivors [2]. However, patients may experience several adverse effects during the course of and after treatment [3]. The negative effect of chemotherapy on the cardiovascular system is a well-known issue [4]. Among the comorbidities and adverse health effects associated with breast cancer are those associated with lipids metabolism. Available evidence suggests that BC survivors are at increased risk for the development of cardiovascular disease [5], metabolic syndrome [6, 7], diabetes and/or dyslipidemia [8, 9]. These come either as a direct result of cancer treatment or due to and in combination with factors such as obesity, weight gain and nutritional factors, physical activity levels or age [10–13], all of which can influence mortality in BC survivors [14] Chemotherapy seems to worsen dyslipidemia in breast cancer patients [15, 16]. In contrast, tamoxifen treatment [17] and radiotherapy [18] were suggested to have a moderate beneficial effect on serum lipids in breast cancer patients.

Alteration in lipid metabolism is a known feature of cancer cells. The frequent association of obesity and overweight with an elevated risk of breast cancer, observed in epidemiological studies, highlighted the notion that adipose tissue and adipocytes interact with, and greatly influence the tumor microenvironment [19]. Although most of the attention so far has been given to enhanced de novo fatty acid (FA) synthesis in cancer cells, the exogenous lipids utilization for energy in cancer cells and oncogenic lipid signaling molecules, show the importance of adipose tissue and dietary intake of lipids in cancer [19, 20].

Most of the available evidence of lipid disturbances observed in BC survivors focuses on concentrations of triacylglycerols (TGs), cholesterol and its fractions [7, 15–18, 21]. However, lipids are a diverse class of compounds, each with unique properties and physiological roles. FAs serve as building blocks for complex lipids and influence their hydrophobicity, depending on the number of fatty acyl chains in lipid molecule, FA length and their degree of saturation. Different backbones of glycerolipids, sphingolipids, glycerophospholipids, and saccharolipids confer further unique properties to these classes, making them more or less amphiphilic. Generally, lipids serve as membrane components, energy sources and storage and have a role in molecular signaling. The length of the Fas’ acyl chain modulates the functional roles of lipids. For example, the presence of methyl branches affects membrane fluidity and permeability [22], the chain length in ceramide species changes their properties from pro-apoptotic to antiapoptotic [23], and the location of double bonds determine pro- or anti-inflammatory properties of the FA metabolites [24] The identification of specific FA provided useful insight into therapeutic strategies or biomarker identification in lung [25], colorectal [26, 27] or breast [28] cancer. Lastly, there is an increased interest in the health benefits of underreported FA classes, namely branched-chain FAs (BCFAs) [22] and odd-chain FAs (OCFAs) [29] in cancer patients.

Lifestyle interventions such as dietary guidance and/or exercise regimen can improve the quality of life [30]. FA alterations after BC treatment may have an impact on the general health and/or the disease recurrence. BC survivors are at increased risk of cardiovascular events [31], diabetes [9], inflammation and chronic fatigue [32]. These adverse effects are in turn frequently associated with specific FAs/FA groups [33–35] and could be potentially addressed with nutritional changes or supplementation of FAs. But providing reliable guidelines for lipid intervention, requires a clearer picture of specific FAs in the serum of BC survivors. This study aimed to characterize the changes in the FA composition of BC survivors and to identify FA disturbances that might be addressed either with therapeutic or dietary interventions.

## Methods

### Patients

The study was conducted according to the guidelines of the Declaration of Helsinki and approved by Independent Bioethics Committee for Scientific Research at the Medical University of Gdansk, Poland (protocol code: NKBBN/526/2013). Informed consent has been obtained from the patients. The control group (*n* = 25) were healthy females with a mean age of 44 ± 10 years. We have recruited patients from oncological out-patients’ clinics in the Pomeranian region of Poland with a histopathologically confirmed breast carcinoma (according to a thick needle biopsy, clinical stage I- III), who were referred to the Department of the Surgical Oncology Medical University of Gdansk. Before the operation, clinical and pathological data such as age, histological type of breast cancer and clinical staging according to TNM classification, presence/lack of estrogen or progesterone receptors were collected. We included patients that were qualified for the following operations: breast-conserving therapy with sentinel lymph node, breast-conserving therapy with auxiliary lymph node dissection, and radically modified breast amputation with or without immediate breast reconstruction. After the operation, the following data were collected: pathological TNM classification, including in particular immunohistochemical results of the tumor. All patients were treated according to current world therapeutic standards both by using neoadjuvant therapy or by adjuvant therapy, depending on the stage of cancer. Five patients received neoadjuvant chemotherapy (paclitaxel/carboplatin); eight patients received neoadjuvant radiotherapy, which lasted up to six months post-surgery. Endocrine therapy regimens (tamoxifen) also ended at the longest at 6 months post-surgery with an exception of two HER + patients, for whom the tamoxifen + herceptin treatment duration was 5 years after the surgery. The clinical characteristics of patients are gathered in Table 1. Most of the patients included in this study presented with ER/PR-positive HER2-negative tumors, which reflects the commonality of this subtype in the overall population. The follow-up serum could not be obtained from 1 patient at the 12-month point (12 M follow-up), and 9 patients at 24 months (24 M follow-up). Patients were not specifically advised to use fish oil or similar supplements.

**Table 1:** Characteristics of breast cancer patients before, 12, and 24 months after treatment

Parameter	Preoperative *n* = 28	12 M follow-up *n* = 27	24 M follow-up *n* = 19
Age [years]	56 ± 11	56 ± 12	56 ± 11
Stage [number of patients]			
I	14	14	11
II	11	10	6
III	2	2	1
unknown	1	1	1
Histological type [number of patients]			
Ductal	21	20	14
Lobular	3	3	3
Papillary	1	1	1
unknown	3	3	1
Involved lymph nodes [%]^a^	12.5 ± 23.6	9 ± 15.8	13.2 ± 26
Number of tumor foci	1.3 ± 0.6	1.3 ± 0.6	1.4 ± 0.7
Expression of estrogen receptors [%]^a^	66.9 ± 41	66.5 ± 41.9	81.9 ± 29.2
Expression of progesterone receptors [%]^a^	41.9 ± 40.7	39.9 ± 40.3	54.8 ± 41.9
ER + , PR + , HER2-	19	18	16
ER-, PR-, HER2 +	0	0	0
TNBC (ER-, PR-, HER2-)	5	5	1
ER + , PR-, HER2-	1	1	1
TPBC (ER + , PR + , HER2 +)	2	2	0

Results are mean ± SD

*ER* estrogen receptor, *HER2* human epidermal growth factor receptor 2, *PR* progesterone receptor, *TNBC* triple-negative breast cancer

^a^The expression of estrogen, progesterone receptors and involved lymph nodes are presented as a percentage of a positive result (cells or tissues) in clinical trials

### Determination of lipidogram in patients

Lipid profile, including total cholesterol (TC), TG, HDL-C, and LDL-C parameters were measured in patient serum using an Automated Photometer (ERBA XL-180, Erba Diagnostics Mannheim Gmbh, Mannheim, Germany) and specific ERBA kits according to the manufacturer’s instructions. Non-HDL-C was calculated by subtraction of HDL-C concentration from TC.

### GC–MS analysis of serum fatty acids

Total lipid were extracted from 200 µl aliquots of serum with the chloroform–methanol mixture following the method of Folch et al. [36]. The lipids were then hydrolyzed by incubation at 90 °C for 3 h with KOH in methanol and FAs were extracted with water/n-hexane. The FA methyl esters (FAMEs) were obtained via the derivatization with 10% boron trifluoride-methanol solution for 1.5 h at 55 °C, and after addition of 1 mL H_2_O to reaction mixture the FAMEs were extracted thrice with n-hexane, solvent was evaporated under nitrogen stream and samples were stored at -20 °C until analysis.

The FAMEs were analyzed with GC-EI-MS QP-2010SE (Shimadzu, Kyoto, Japan). The separation was achieved on Zebron ZB-5MSi capillary column (30 m length × 0.25 mm i.d. × 0.25 μm film thickness). Samples were injected in dichloromethane. The GC oven temperature was set at 60–310 °C (4 °C/ min, 5 min hold at 310 °C) with overall run time of 67.5 min. The carrier gas applied was helium at column head pressure of 100 kPa. Mass spectrometry detection was conducted in full scan mode, with the mass scan range set at m/z 45–700 with an electron impact source operating at 70 eV. 19-methylarachidic acid was used as an internal standard. FAs identification was aided by the standards reference mixture (37 FAME Mix, Sigma-Aldrich) and reference library NIST 2011.

### Univariate and multivariate analysis

The statistical significance of the differences for tested parameters of preoperative group vs 12 M follow-up, preoperative group vs 24 M follow-up, and 12 M follow-up vs 24 M follow-up were verified with a paired t-Student’s test for data with normal distribution, and a Wilcoxon Signed Rank Test for non-parametric data. For multiple group comparison One Way Analysis of Variance (ANOVA) was performed with all pairwise multiple comparison procedure—Tukey Test for parametric data and Kruskal–Wallis ANOVA on ranks followed by all pairwise multiple comparison procedure using Dunn's Method for data with non-normal distribution. Exclusion of data followed the 1.5*IQR (interquartile) rule. All statistical tests were performed with the significance level of α = 0.05 and results were deemed significant if power of applied test was above 0.800. A priori calculated ANOVA power was 0.897. Results are given as mean ± standard error of the mean (SEM). Spearman's Rank or Pearson correlation coefficient was calculated. All univariate calculations were carried out with SigmaPlot software (Systat, Software Inc., San Jose, CA, USA).

The multivariate data analysis was performed with SIMCA software (version 16 Sartorius Stedim Data Analytics AB, Umeå, Sweden). Pareto scaling was applied to data and for skewed variables log transformation was performed. The unsupervised Principal Component Analysis (PCA) was performed to reveal natural clustering of samples. The PCA biplot was constructed from the first two components, with Hotelling’s T2 range of 95% applied. Supervised, partial least squares analysis (PLS-DA) was performed on significant variables selected based on the results of paired t-tests, variables with *p* < 0.05 were chosen. The PLS-DA models underwent cross-validation analysis of variance (CV-ANOVA) to assess their reliability. The variables most important in PLS-DA analysis were those with variable importance score (VIP) above 1.0. For both PCA and PLS-DA models variables with > 50% missing values (FAs that were detected in trace amounts) were excluded from the analysis.

## Results

The blood lipid profile analysis showed no significant differences in TC and LDL-C concentrations across study groups (Table 2). Patients with breast cancer displayed significantly elevated serum TG concentrations when compared to the control group at all time points (Table 2). Also, the breast cancer patients were characterized by lower HDL-C concentrations in the blood than the control group, which was significant at 12 M and 24 M follow-up.

**Table 2:** Lipidogram of study subjects

	**Serum concentration**				***p*-value for comparison**					
**Parameter**	**Control** ***n* = 25**	**Preoperative** ***n* = 28**	**12 M follow-up** ***n* = 27**	**24 M follow-up** ***n* = 19**	**Pre vs Control**	**12 M vs Control**	**24 M vs Control**	12 M vs Pre	24 M vs Pre	12 M vs 24 M
TC [mg/dL]	184 ± 12.0	196 ± 7.58	186 ± 7.84	181 ± 5.95	NS	NS	NS	NS	NS	NS
TG [mg/dL]	108 ± 13.4	163 ± 18.8	174 ± 18.3	177 ± 24.5	< 0.05*	< 0.05*	< 0.05*	NS	NS	NS
HDL-C [mg/dL]	51.5 ± 2.92	43.1 ± 2.30	39.8 ± 2.12	40.7 ± 7.23	0.051	0.003	0.017	NS	NS	NS
LDL-C [mg/dL]	106 ± 8.03	124 ± 6.97	117 ± 6.77	117 ± 5.93	NS	NS	NS	NS	NS	NS
non-HDL-C [mg/dL]^a^	132 ± 10.1	152 ± 7.81	146 ± 8.01	140 ± 5.77	NS	NS	NS	NS	NS	NS

Mean ± SEM; *p*-value from All Pairwise Multiple Comparison Procedures (Tukey Test)

*NS* not significant, *HDL-C* high-density lipoprotein cholesterol, *LDL-C* low density lipoprotein cholesterol, *Pre* preoperative, *TC* total cholesterol, *TG* triacylglycerols

^*^ *p*-value from Kruskal–Wallis One Way Analysis of Variance on Ranks followed by All Pairwise Multiple Comparison Procedures (Dunn's Method)

^a^[non-HDL-C] = [TC] – [HDL-C]

**Fig. 1:**
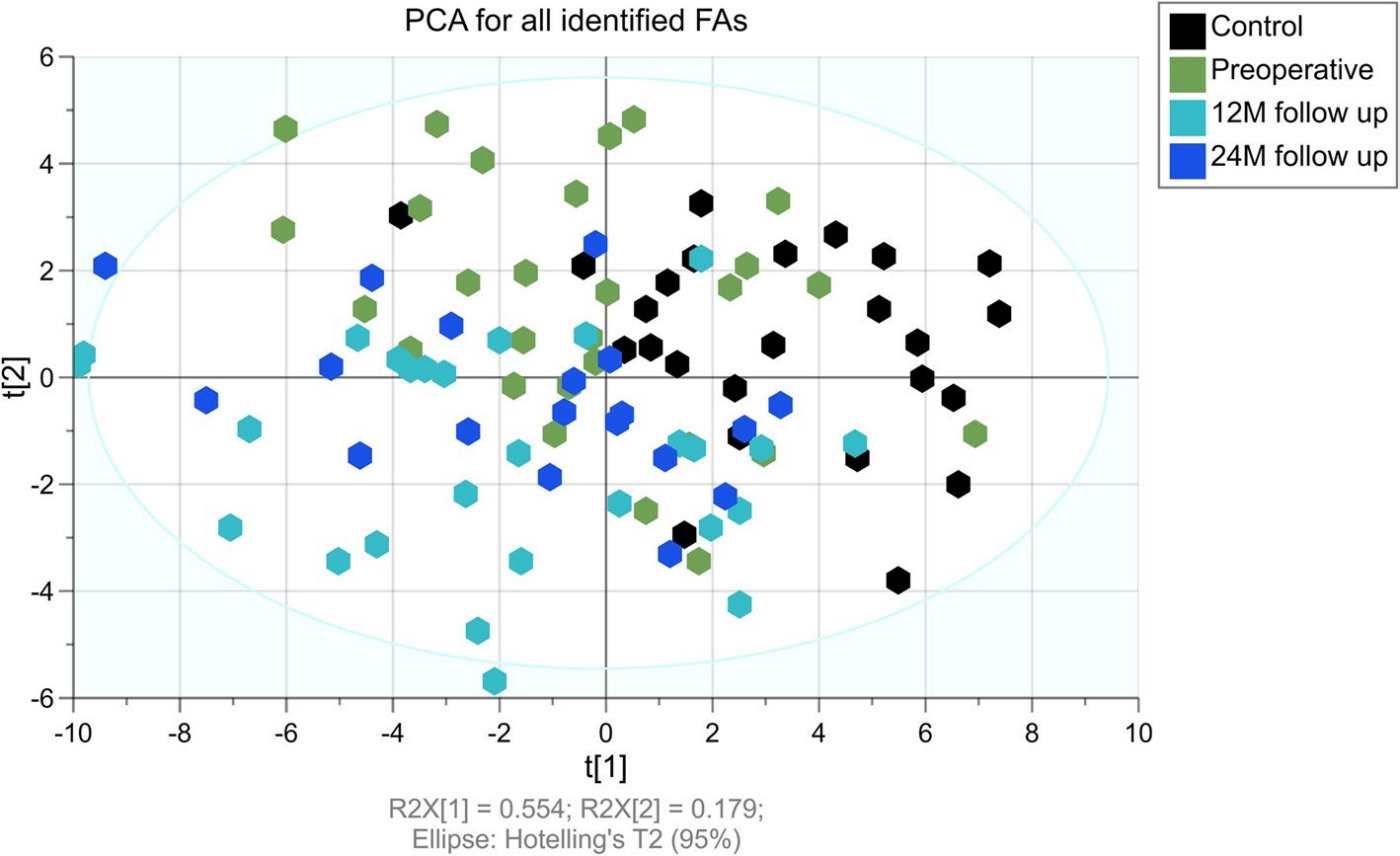
The results of principal component analysis based on whole fatty acid profiles in serum of breast cancer patients at different stages of therapy and healthy controls

**Fig. 2:**
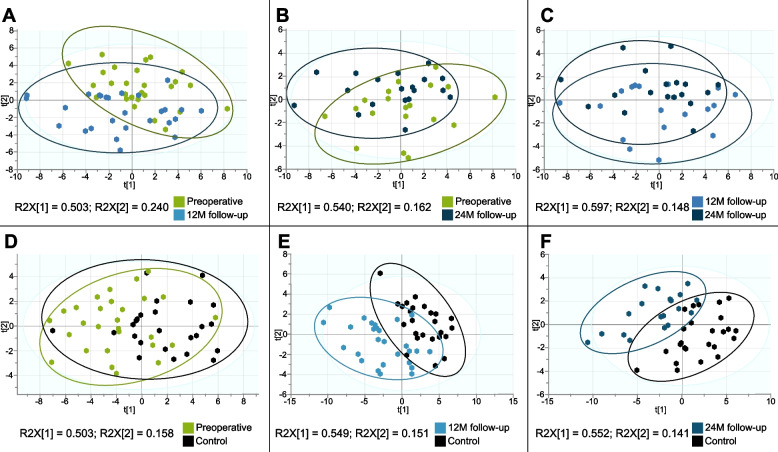
Unsupervised principal component analysis (PCA) for two group comparisons. Models including whole serum FA profiles from (**A**) preoperative patients and 12 M follow-up (*n* = 27), **B** preoperative patients and 24 M follow-up (*n* = 19), **C** patients at 12 M follow-up and 24 M follow-up (*n* = 18), **D** preoperative patients (*n* = 28) and controls (*n* = 25), **E** patients at 12 M follow-up (*n* = 27) and controls (*n* = 25), **F** patients at 24 M follow-up (*n* = 19) and controls (*n* = 25)

The unsupervised multivariate analysis by PCA, which included all measured FAs as variables, was performed to verify the natural clustering of the study subject’s groups based on serum FA profiles (Fig. 1). The resulting two-component model accounted for 74.3% variability in the samples and showed a large scattering of study subjects without clear grouping trends across PC1 and PC2. This indicates heterogeneity in FA profiles of BC survivors. The PCA model summary is given in Table S1. Subsequently, to consider differences between patients’ FA profiles at different periods after surgery, the PCA models were built for paired samples from patients before operation and at 12 M follow-up (Fig. 2A), before operation and at 24 M follow-up (Fig. 2B) and at 12 M and 24 M follow-up (Fig. 2C). The highest tendency to separate was observed for comparison between preoperative and 12 M follow-up patients (84.4% of total variance across PC1 and PC2), however, the separation was not complete. The FA profiles measured at 12 M and 24 M clustered together, with no apparent grouping trends. Similarly, the PCA models were built for breast cancer patients before, 12 and 24 months after the surgery and the control group (Fig. 2D-F). The 12 M follow-up (Fig. 2E) and 24 M follow-up (Fig. 2F) groups exhibited clearer separation from control subjects than preoperative patients from controls (Fig. 2D). Taken together, this results show that at 12 months serum FA profiles of BC patients are most dissimilar to both preoperative and control profiles.

**Fig. 3:**
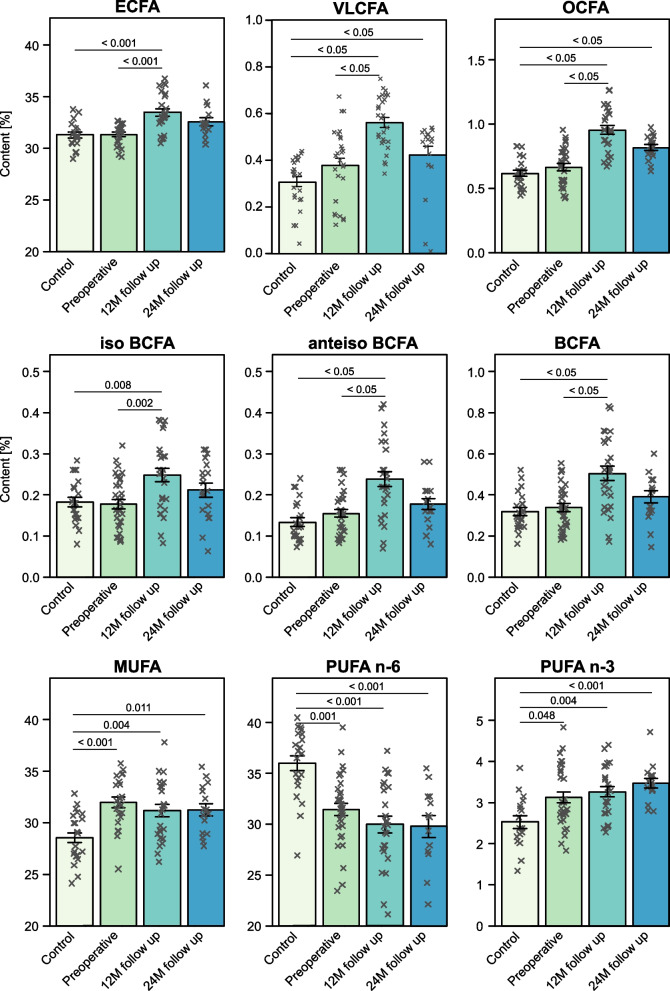
The serum fatty acid content [%] of main fatty acid groups in the serum of study subjects. *p*-values from All Pairwise Multiple Comparison Procedures (Tukey Test) or from Kruskal–Wallis One Way Analysis of Variance on Ranks followed by All Pairwise Multiple Comparison Procedures (Dunn's Method). BCFA: branched chain fatty acids; ECFA: even chain saturated fatty acids; MUFA: monounsaturated fatty acids; OCFA: odd chain saturated fatty acids; PUFA: polyunsaturated fatty acids; VLCFA – very long chain saturated fatty acids with > 20 carbons in acyl chain

When considering the effect of the treatment of breast cancer patients on the total contents of the main groups of FA, we observed the largest number of differences at 12 M follow-up compared to preoperative results. We found increased levels of even chain FAs (ECFA), OCFA, BCFA and the sum of all saturated FAs (SFAs) 12 months after surgery (Fig. 3). After 24 months we did not find any significant changes in any FA group compared to 12 M or preoperative results. The results for specific FAs from studied groups are presented in Table S2, and confirmed that the most numerous differences among specific FA were at 12 M follow-up when compared to preoperative results. Since the control group and BC patients differed in age, we have additionally reduced the control group to the population of 18 subjects whose age was not significantly different from BC patients, to verify if the age might significantly contribute to the FA differences The comparison of FA composition within these groups has been presented in Table S3 in the revised manuscript. Still, comparing FA profiles in BC patients to this limited control group have led to similar conclusions, thus we decided to use the entire control group for analyzes to increase the power of the tests.

When comparing breast cancer patients to controls, the preoperative breast cancer patients were characterized by elevated monounsaturated FAs (MUFAs) and n-3 PUFAs content, whereas the n-6 PUFAs were significantly decreased when compared to control serum (Fig. 3). Essential n-6 PUFA – linoleic acid (LA, 18:2 n-6) serum content as well as n-6/n-3 ratio was significantly lower in preoperative breast cancer patients when compared to the control group (Table S2). Levels of other significantly different PUFAs—20:2 n-6 and long-chain n-3 PUFAs – eicosapentaenoic acid (EPA, 20:5 n-3) and docosapentaenoic acid (DPA, 22:5 n-3) were higher in preoperative breast cancer patients when compared to controls (Table S2). The differences described above persisted in the breast cancer patients 12 and 24 months after breast cancer resection (Fig. 3 and Table S2). More differences were identified when comparing control subjects with patients at 12 M and 24 M follow-ups, including (in addition to those listed above) increased ECFA (12 M), OCFA (12 M, 24 M), and BCFA (12 M) (Fig. 3). This trend can be also observed when analyzing the specific FA from these groups (Table S2). Interestingly, we were able to detect a higher amount of very long chain FAs (VLCFAs, C > 20) in the serum of breast cancer patients 12 months after surgery comparing them both to healthy subjects and their preoperative results (Fig. 3).

The analysis of a wide FA profile in serum allows for the estimation of the whole-body activity of enzymes which metabolize FAs by calculating the ratios of products to substrates. The stearoyl-CoA desaturase-1 (SCD-1) activity index was assessed as an 18:1 to 18:0 ratio and showed significantly higher activity in patients before treatment when compared to both the control group and patients in follow-up (Table S2). The delta-5 desaturase activity was assessed using the ratio of arachidonic (ARA, 20:4 n-6) to dihomo-γ-linolenic acid (DGLA, 20:3 n-6) content and was lower in breast cancer patients than in the control group, albeit non-significantly, and there was a trend to decrease in patients in subsequent follow-ups (Table S2). Conversely, the delta-6 desaturase index, calculated as DGLA to LA ratio, was the lowest in the control group and tended to increase in breast cancer patients in follow-ups (Table S2).

**Fig. 4:**
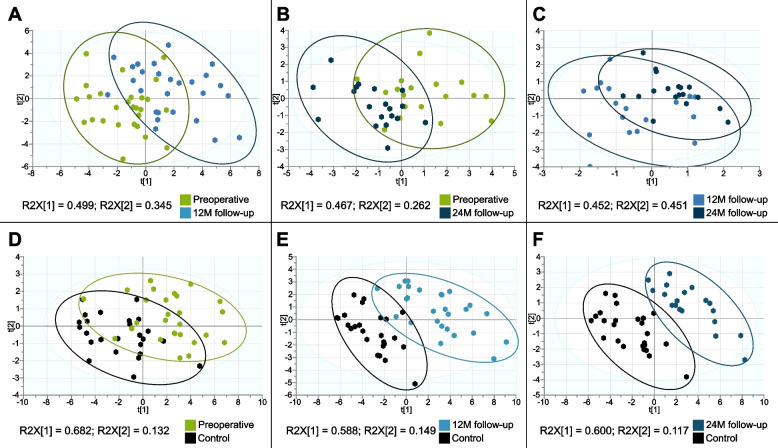
Results from partial least squares supervised analysis (PLS-DA). Models built using significantly different serum fatty acids in (**A**) preoperative patients and 12 M follow-up (*n* = 27), **B** preoperative patients and 24 M follow-up (*n* = 19), **C** patients at 12 M and 24 M follow-up (*n* = 18), **D** preoperative patients (*n* = 28) and control (*n* = 25), **E** patients at 12 M follow-up (*n* = 27) and controls (*n* = 25), **F** patients at 24 M follow-up (*n* = 19) and controls (*n* = 25). Fatty acids included as variables were selected based on significance in paired t-tests for comparisons between preoperative patients and follow-ups (see Tables S4, S5 and S6 respectively) or two-way t-Student’s test for comparisons between breast cancer patients and controls (Table S8)

Additionally, since in the unsupervised PCA analysis did not allow for grouping of patients based on whole FA serum profiles (Figs. 1 and 2A-C), the supervised PLS-DA was performed for comparison between each two groups of patients. The variables for this analysis were selected based on the significance (*p*-values) of paired t-tests performed for each comparison (Tables S4-S6), and the most promising FAs/FA groups, which differed statistically between compared groups (*p* < 0.05) were included. This type of analysis allows also for the identification of parameters with the highest impact on the separation of the groups of data based on variable importance in projection (VIP) scores. Similarly, to PCA models, the best separation in PLS-DA models was observed for comparison between preoperative and 12 M follow-up patients (Fig. 4A), differences were also observed for the preoperative vs 24 M follow-up model (Fig. 4B); however, the PLS-DA model for 12 M vs 24 M follow-up (Fig. 4C) comparison did not pass the ANOVA cross validation (Table S7). The cross-validated R^2^ values obtained for the PLS-DA models indicated a good description of data, but the predictability of these models is poor (Q^2^ < 0.4) (Table S7).

The PLS-DA models were also built using variables that differed significantly between the control group and breast cancer patients (Table S8). The PLS-DA analysis allowed for better separation between preoperative patients and the control group (Fig. 4D), however, the predictive value of the model was non-satisfactory (Q^2^ = 0.411) (Table S7). This model revealed good separation between controls and patients at 12 M and 24 M follow up (Fig. 4E, F) with high Q^2^ (0.815 and 0.847 respectively) (Table S7).

For significant PLS-DA models (comparisons between preoperative vs 12 M, preoperative vs 24 M, control vs preoperative, control vs 12 M and control vs 24 M) the VIP scores was used to assess the importance of the given variable in variance explanation for each analysis, and the percentage contents for FAs/FA groups with VIP scores above 1.00 are shown in Figure S1.

## Discussion

The main finding of this study is the lack of normalization of the FA profiles in the serum of BC patients after breast cancer resection. Even 24 months after surgery patients exhibited altered levels of long-chain SFAs/MUFAs and still heightened levels of most analyzed PUFAs (Table S2). The patients' serum FA contents were markedly different from those of the healthy control group at both follow-up time points as evidenced by the number of differences in individual FA levels (Table S2) and separation of groups in unsupervised PCA models (Figs. 1 and 2) as well as in PLS-DA analysis (Fig. 4). The differences in FA profiles between healthy control subjects and patients 12 and 24 months after tumor resection were more pronounced than between controls and patients before surgery. The supervised PLS-DA analysis allowed us to identify FA with the highest impact on the separation. These were 18:0, 14:0, 18:1, 20:1 and LA, for models differentiating BC patients before resection and in subsequent follow-ups (Figure S1A and B). Additionally, EPA and docosahexaenoic acid (DHA, 22:6 n-3) proved to be important in separation between breast cancer patients and healthy control subjects (Figure S1C and E). The differences in FA profiles between healthy subjects and BC patients and the changes found between stages of treatment, may be caused by modification of lifestyle factors, different expression patterns of enzymes involved in lipid metabolism and used therapy, however, such analysis was beyond the aims of this study and requires further research.

In past years numerous studies suggested the association between blood lipid profile and increased risk of various cancers [37]. In this study, the patients’ blood lipid profile was unfavorable [38] at baseline when compared to the control group, and did not change significantly during at 24 M. Our patients presented with higher TG and lower HDL-C. The role of lipoproteins in BC development is still debated, with studies showing divergent results [39–41]. One meta-analysis found an inverse correlation between serum TG levels and BC risk; however, this association seems to disappear when adjusted for major dietary factors [39]. Another meta-analysis revealed higher TG and lower HDL levels in breast cancer patients compared with healthy controls [40], which is in line with our results. HDL cholesterol and apolipoprotein A1, were indicated to be involved in tumorigenesis through the regulation of proliferative and inflammatory pathways [42]. Tian et al. reported that chemotherapy impacts negatively TG, LDL and HDL values, with high TG levels persisting six months after therapy completion [43]. Another study reported that dyslipidemia persists even up to 12 months [16]. However, Arpino et al. suggested that the observed changes in blood lipid profile may be merely a result of lifestyle changes following diagnosis rather than a consequence of the therapeutic regimen [44]. Nevertheless, it seems reasonable to introduce appropriate treatment in dyslipidemic breast cancer patients to normalize their lipidogram.

Enhanced de novo lipogenesis emerged as a hallmark of many types of cancer, especially those associated with obesity [19, 45, 46]. Studies report upregulated fatty acid synthase (FASN) expression in cancer and pre-cancer cells [47–49]. Since we did not directly evaluate FASN expression in the cancer tissue of patients, we cannot conclude that FASN activity is changed in preoperative BC patients. Contrary to some studies [48, 50, 51], we did not find increased levels of blood SFAs in preoperative patients when compared to the control group. However, SFA levels were increased in 12 M follow-up compared to preoperative results. A meta-analysis conducted by Brennan et al. [52] suggests that a high intake of saturated fat is associated with higher breast cancer-specific death. In primary mouse embryonic fibroblasts SFAs were shown to have a negative influence on the DNA damage response pathway, possibly promoting cell transformation and contributing to tumor progression and growth [49]. Xu et al. [48] reported that FASN overexpression may promote tumor development and enhance cancer cell proliferation by providing FAs for membrane formation; and migration by increasing levels of SFAs involved in lipid signal transduction. Higher levels of SFAs due to dietary intake may have a similar effect. Thus, increased SFA 12 M after surgery in BC patients may potentially increase the risk of the recurrence of the disease, and reduction of SFA intake may constitute a therapeutic target for patients during treatment.

A surprising result of this study is elevated levels of saturated VLCFAs sub-group in follow-up patients when compared to baseline, with differences most pronounced in 12 M follow-up. VLCFAs are highly hydrophobic and confer unique properties, altering membranes fluidity, permeability, lipid microdomain formation or clustering [53]. Acyl chain elongation has also been identified as a potential diagnostic trait [26] or target for treatment [25] in different cancers. We did not detect significant differences, only a trend to increase, of circulating VLCFAs in preoperative patients compared to controls. However, after 12 months serum VLCFA were significantly increased in breast cancer patients compared to preoperative results.

In recent years BCFAs have been recognized as an underexplored bioactive FA class. They sparked a growing research interest due to their potential beneficial effects on health in obesity [54], anti-inflammatory effects [55], glucose metabolism maintenance [56] and anti-cancer activity [57–60]. The main sources of BCFAs are dairy products and ruminant meats, although humans are capable of endogenous synthesis [22]. Incorporation of BCFAs into cell membranes can modulate their fluidity and lead to disruption of membrane integrity, cell dysfunction and death. The magnitude of this effect is determined by the structure of BCFA, with iso-BCFAs showing greater cytotoxicity than anteiso-BCFAs [59]. Iso-BCFAs show cytotoxicity towards BC cell lines, with iso-16:0 exhibiting the highest activity [58]. In SKBR-3 breast cancer cells, iso-15:0 was shown to incorporate into glycerophospholipids and trigger apoptosis [57], that may protect patients from disease recurrence. We observed an increase in iso-15:0 serum content in 12 M follow-up. However, after 24 months its levels returned to preoperative values. Moreover, the level of iso-series BCFAs in our study was negatively correlated with serum TG in breast cancer patients at baseline (-0.434 at *p* < 0.024). We previously observed a similar association between serum BCFAs and TG in bariatric patients [54, 61], supporting the idea that BCFAs are involved in the regulation of fat storage. The 2015 meta-analysis showed that high dairy consumption was linked to reduced BC risk [62]. To the best of our knowledge, our study is the first to report the levels of serum BCFAs in breast cancer patients. A similar scarcity of data exists for another bioactive group of dairy-derived FAs – OCFAs. Recently, there has been increased interest in OCFAs due to their association with reduced risk for coronary heart disease and type II diabetes [29, 61]. The data on OCFAs role in cancer pathology is extremely limited. Thus far cell studies indicated an inhibitory effect on proliferation of 19:0 in hemo-lymphocytic cancer [63], 17:0 in non-small cell lung carcinoma [64] and suppression of migratory and invasive capability of breast cancer stem cells by 15:0 [65]. Collectively, increased levels of OCFAs and BCFA found in the serum of BC patients in this study at the time of follow-ups, may represent beneficial change and perhaps even protect BC recurrence.

PUFAs have a pleiotropic effect on health due to their involvement in inflammation control via the generation of potent pro- and anti-inflammatory metabolites [24]. In this study, we observed a significant decrease in serum levels of LA, an endogenous precursor of n-6 series PUFAs in humans, in preoperative BC patients when compared to the control group and even lower, in subsequent follow-ups. Low preoperative n-6 PUFAs in BC patients are an unexpected result, since cancer patients frequently present with higher or unchanged levels when compared to healthy controls [51, 66–69]. BC patients also presented with unusually high n-3 PUFAs content. We were not able to directly asses patients’ diets, however, a high proportion of BC patients makes voluntary changes in their eating habits, particularly by reducing the consumption of fats and fatty food [70]. This could account for proportions of PUFAs as well MUFAs content. Yamashita et al. [28] reported that the levels of LA were significantly decreased in tumor tissue when compared to corresponding normal breast tissue. Tomida et al. [71] reported that the ratio of 18:2- to 18:1-containing lipid species in BC patients’ serum was significantly decreased before surgery, which is consistent with our findings (Table S2). However, unlike in the previous study [71], 18:2/18:1 ratio did not normalize. The low levels of LA in BC patients during therapy might be linked to enhanced production of oxylipins. The analysis by Chocholoušková et. al. [72] revealed that in BC patients LA-derived octadecanoids, i.e. 9-HODE and 13-HODE, are heavily upregulated. We have also observed a trend of increased levels of eicosatetraenoic acid (ETA, 20:4 n-3) and DHA during the treatment of BC patients. These trends were similar to those reported before [48, 51]. Studies examining the protective effects of dietary n-3 PUFAs (EPA and DHA) against mammary carcinogenesis largely suggest that they are beneficial for reducing the BC risk [24]. Although we did not observe significant changes in EPA and DHA in patients during cancer treatment, their levels were elevated when compared with the control group. A study assessing the n-3 PUFAs intake in early-stage breast cancer survivors participating in the Women’s Healthy Eating and Living (WHEL) study indicated that dietary n-3 PUFA intake is associated with reduced risk of all-cause mortality as well as recurrence or development of new invasive breast cancer [73]. There is also an ongoing clinical trial assessing the effect of supplementation of EPA + DHA in breast cancer survivors [74]. The rationale behind increasing dietary n-3 PUFAs in breast cancer patients is to decrease risk [75, 76] and improve outcomes [77] by elevating the levels of anti-inflammatory n-3 PUFA metabolites relative to n-6 PUFA metabolites. Therefore, the decreased n-6/n-3 PUFA ratio in the serum of breast cancer patients at follow-up may represent a beneficial trend. Higher n-3 PUFA consumption might be therefore favorable for BC outcomes for example due to the cardioprotective benefits [24, 77].

## Conclusions

**Fig. 5:**
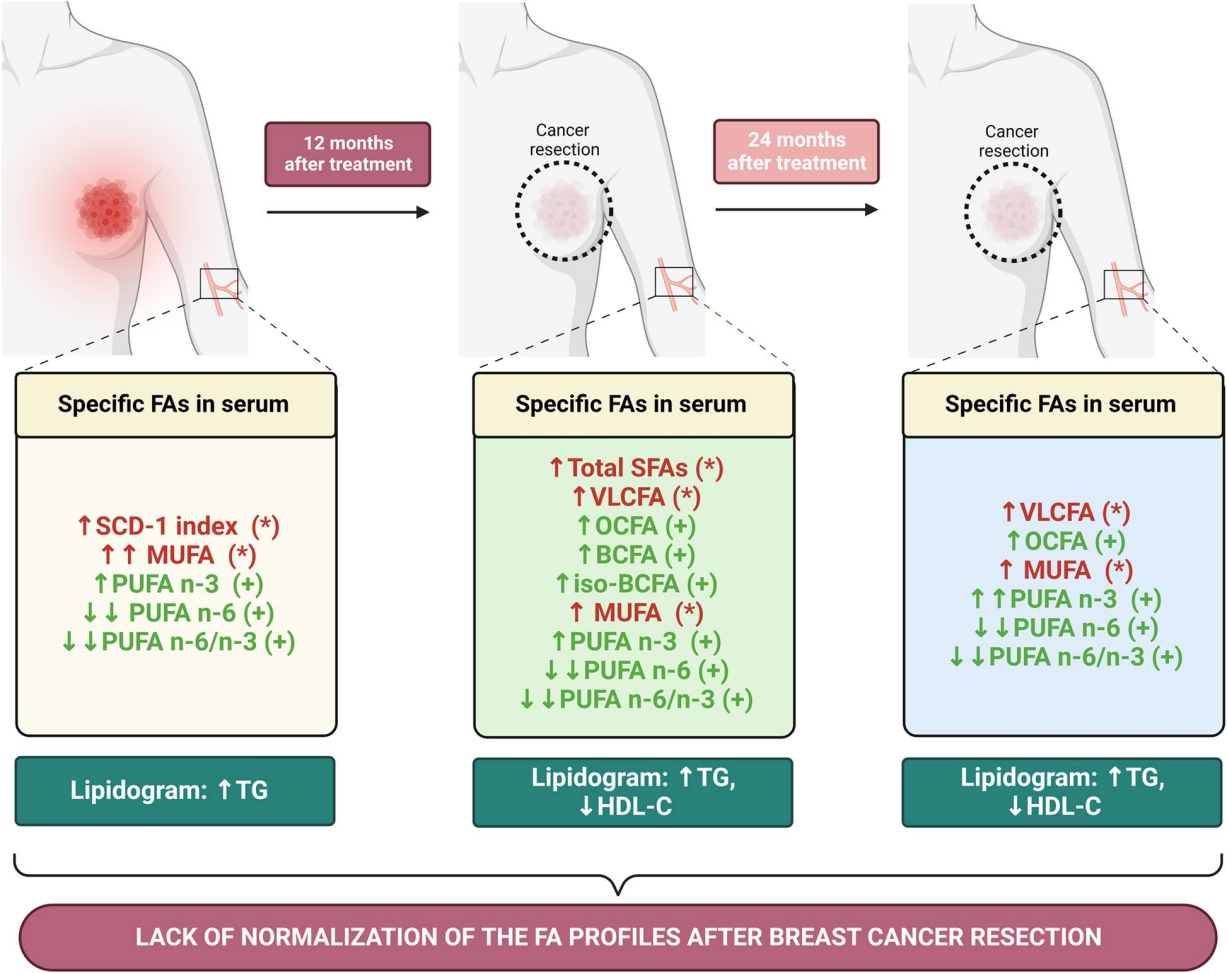
Alteration in selected classes of FA in breast cancer survivors suggests a therapeutic opportunity for suppressing cancer recurrence. Arrows up (↑:*p* < 0.05; ↑↑; *p* < 0.001) refer to a significantly higher concentration of FA and arrows down (↑:*p* < 0.05; ↓↓: *p* < 0.001) are related to a decreased level of FA, compared to healthy control. Mark (*) represents a contribution to tumor progression and ( +) antitumor properties of specific FA. Created with BioRender.com (accessed on 31 October 2022)

In this study we have found significant differences in serum levels of FAs in BC patients before tumor removal and at 12- and 24-months follow-up (summarized in Fig. 5). Many of those changes appear to be potentially beneficial, like increases in OCFA and BCFA or lowered n-6/n-3 PUFA ratio, some may be unfavorable, like increased SFA, whereas others, such as increased VLCFAs, warrant further explanation. The limitations of our study include the relatively small cohort and the fact that we were not able to assess the influence of diet on FA profiles due to the lack of nutrition data. Nonetheless, this study concerns the little-studied issue of serum FA profile changes after breast tumor resection and provides novel data on previously underreported groups of FAs – BCFAs and OCFAs. Additional studies on the link between altered FA profiles and BC survival would help to form dietary recommendations for patients after BC resection.

## Supplementary Information

**Additional file 1:** **Table S1.** Principal component analysis (PCA) models summary. **Table S2.** Comparison of serum fatty acid profiles between the control group and breast cancer patients preoperative and in follow-ups. **Table S3.** Comparison of serum fatty acid profiles between the age-matched control group and breast cancer patients preoperative and in follow-ups. **Table S4.** Comparison of fatty acid serum content in paired samples from preoperative patients and 12 months follow-up. **Table S5.** Comparison of fatty acid serum content in paired samples from preoperative patients and 24 months follow-up. **Table S6.** Comparison of fatty acid serum content in paired samples from patients in 12 months and 24 months follow-up. **Table S7.** Discriminant analysis models summary. **Table S8.** *P*-values from two-way t-Student’s test for comparisons between breast cancer patients’ serum fatty acids levels at different time points versus the control group. **Figure S1.** Boxplots for variables with VIP scores above 1 in significant PLS-DA models.

## Data Availability

The datasets generated during the current study are available from the corresponding author on reasonable request.

## Abbreviations

ANOVA: Analysis of variance
ARA: Arachidonic acid
BCFA: Branched-chain fatty acid
DGLA: Dihomo-γ-linolenic acid
DHA: Docosahexaenoic fatty acid
ECFA: Even-chain fatty acid
ELOVL: Fatty acid elongase
EPA: Eicosapentaenoic fatty acid
ER: Estrogen
FA: Fatty acid
FAME: Fatty acid methyl ester
FASN: Fatty acid synthase
HDL: High density lipoprotein
HER2: Human epidermal growth factor receptor 2
LA: Linoleic acid
LDL: Low density lipoprotein
MUFA: Monounsaturated fatty acid
OCFA: Odd-chain fatty acid
PCA: Principal component analysis
PLS-DA: Partial least squares discriminant analysis
PR: Progesterone
PUFA: Polyunsaturated fatty acid
SCD-1: Stearoyl-CoA desaturase-1
SFA: Saturated fatty acid
TC: Total cholesterol
TG: Triglycerides
VIP: Variable importance projection
VLCFA: Very long chain fatty acid
