# Supplementary material for: Serum fatty acid profiles in breast cancer patients following treatment
Source: BMC Cancer. 2023 May 12;23:433. doi: 10.1186/s12885-023-10914-2 (PMC10176817; doi:10.1186/s12885-023-10914-2)
Supplement: Supplementary file 1 — Additional file 1: Table S1. Principal component analysis (PCA) models summary. Table S2. Comparison of serum fatty acid profiles between the control group and breast cancer patients preoperative and in follow-ups. Table S3. Comparison of serum fatty acid profiles between the age-matched control group and breast cancer patients preoperative and in follow-ups. Table S4. Comparison of fatty acid serum content in paired samples from preoperative patients and 12 months follow-up. Table S5. Comparison of fatty acid serum content in paired samples from preoperative patients and 24 months follow-up. Table S6. Comparison of fatty acid serum content in paired samples from patients in 12 months and 24 months follow-up. Table S7. Discriminant analysis models summary. Table S8. P-values from two-way t-Student’s test for comparisons between breast cancer patients’ serum fatty acids levels at different time points versus the control group. Figure S1. Boxplots for variables with VIP scores above 1 in significant PLS-DA models. [file 12885_2023_10914_MOESM1_ESM.docx]

Supplementary Material

Serum fatty acid profiles in breast cancer patients following treatment

Alicja Pakiet, Agata Jędrzejewska, Katarzyna Duzowska, Alina Wacławska, Patrycja Jabłońska, Jacek Zieliński, Adriana Mika, Tomasz Śledziński and Ewa Słomińska

Contents:

**Table S1**: Principal component analysis (PCA) models summary.

**Table S2**: Comparison of serum fatty acid profiles between the control group and breast cancer patients preoperative and in follow-ups.

**Table S3**: Comparison of serum fatty acid profiles between the age-matched control group and breast cancer patients preoperative and in follow-ups.

**Table S4**: Comparison of fatty acid serum content in paired samples from preoperative patients and 12 months follow-up.

**Table S5**: Comparison of fatty acid serum content in paired samples from preoperative patients and 24 months follow-up.

**Table S6**: Comparison of fatty acid serum content in paired samples from patients in 12 months and 24 months follow-up.

**Table S7**: Discriminant analysis models summary.

**Table S8**: P-values from two-way t-Student’s test for comparisons between breast cancer patients’ serum fatty acids levels at different time points versus the control group.

**Figure S1**: Boxplots for variables with VIP scores above 1 in significant PLS-DA models.

**Table S1. Principal component analysis (PCA) models summary**.

| **Comparison** | **N** | **PCs** | **R^2^X(cum)** |
| --- | --- | --- | --- |
| All study subjects | 99 | 2 | 0.743 |
| Preoperative vs 12M follow-up | 54 | 2 | 0.743 |
| Preoperative vs 24M follow-up | 38 | 2 | 0.702 |
| 12M follow-up vs 24M follow-up | 36 | 2 | 0.745 |
| Control vs preoperative | 53 | 2 | 0.611 |
| Control vs 12M follow-up | 52 | 2 | 0.700 |
| Control vs 24M follow-up | 44 | 2 | 0.693 |

PC: principal component.

**Table S2. Comparison of serum fatty acid profiles between the control group and breast cancer patients preoperative and in follow-ups.**

|  | **Serum content [%]** | | | | **p-value for comparison** | | | | | |
| --- | --- | --- | --- | --- | --- | --- | --- | --- | --- | --- |
| **Fatty acid** | **Control**  **n = 25** | **Preoperative**  **n = 28** | **12M follow-up**  **n = 27** | **24M follow-up**  **n = 19** | **C vs Pre** | **C vs 12M** | **C vs 24M** | **Pre vs 12M** | **Pre vs 24M** | **12M vs 24M** |
| **Even chain FAs** |  |  |  |  |  |  |  |  |  |  |
| 8:0 | N/D | traces | traces | traces | NS | NS | NS | NS | NS | NS |
| 10:0 | 0.014 ± 0.004 | 0.007 ± 0.001 | 0.004 ± 0.001 | 0.005 ± 0.001 | NS | < 0.05* | NS | NS | NS | NS |
| 12:0 | 0.085 ± 0.012 | 0.114 ± 0.017 | 0.215 ± 0.013 | 0.136 ± 0.016 | NS | < 0.05* | NS | < 0.05* | NS | < 0.05* |
| 14:0 | 1.03 ± 0.075 | 0.989 ± 0.051 | 1.69 ± 0.089 | 1.34 ± 0.061 | NS | < 0.05* | < 0.05* | < 0.05* | < 0.05* | NS |
| 16:0 | 22.9 ± 0.284 | 22.8 ± 0.216 | 22.9 ± 0.298 | 22.7 ± 0.368 | NS | NS | NS | NS | NS | NS |
| 18:0 | 7.20 ± 0.136 | 7.10 ± 0.133 | 7.90 ± 0.127 | 7.90 ± 0.158 | NS | 0.002 | 0.002 | < 0.001 | < 0.001 | NS |
| 20:0 | 0.077 ± 0.004 | 0.116 ± 0.010 | 0.167 ± 0.005 | 0.164 ± 0.006 | NS | < 0.05* | < 0.05* | < 0.05* | < 0.05* | NS |
| 22:0 | 0.172 ± 0.009 | 0.189 ± 0.013 | 0.250 ± 0.009 | 0.235 ± 0.007 | NS | < 0.05* | < 0.05* | < 0.05* | NS | NS |
| 24:0 | 0.138 ± 0.010 | 0.176 ± 0.015 | 0.266 ± 0.012 | 0.224 ± 0.006 | NS | < 0.05* | < 0.05* | < 0.05* | NS | NS |
| 26:0 | 0.014 ± 0.002 | 0.013 ± 0.001 | 0.031 ± 0.003 | 0.017 ± 0.001 | NS | < 0.05* | NS | < 0.05* | NS | < 0.05* |
| 28:0 | traces | traces | 0.013 ± 0.002 | 0.009 ± 0.001 | NS | NS | NS | NS | NS | NS |
| 30:0 | traces | traces | traces | traces | NS | NS | NS | NS | NS | NS |
| **Odd chain FAs** |  |  |  |  |  |  |  |  |  |  |
| 9:0 | N/D | 0.012 ± 0.009 | traces | traces | NS | NS | NS | NS | NS | NS |
| 11:0 | 0.005 ± 0.001 | 0.003 ± 0.000 | 0.003 ± 0.000 | 0.003 ± 0.000 | NS | NS | NS | NS | NS | NS |
| 13:0 | 0.012 ± 0.001 | 0.010 ± 0.001 | 0.011 ± 0.001 | 0.010 ± 0.001 | NS | NS | NS | NS | NS | NS |
| 15:0 | 0.258 ± 0.013 | 0.270 ± 0.012 | 0.388 ± 0.020 | 0.296 ± 0.014 | NS | < 0.05* | NS | < 0.05* | NS | < 0.05* |
| 17:0 | 0.248 ± 0.009 | 0.289 ± 0.013 | 0.353 ± 0.010 | 0.329 ± 0.006 | NS | < 0.05* | < 0.05* | < 0.05* | NS | NS |
| 19:0 | 0.016 ± 0.001 | 0.018 ± 0.001 | 0.032 ± 0.002 | 0.026 ± 0.002 | NS | < 0.05* | < 0.05* | < 0.05* | NS | NS |
| 21:0 | 0.015 ± 0.001 | 0.016 ± 0.002 | 0.027 ± 0.002 | 0.029 ± 0.002 | NS | < 0.05* | < 0.05* | < 0.05* | < 0.05* | NS |
| 23:0 | 0.052 ± 0.003 | 0.073 ± 0.008 | 0.112 ± 0.005 | 0.099 ± 0.006 | NS | < 0.05* | < 0.05* | < 0.05* | NS | NS |
| 25:0 | 0.015 ± 0.002 | 0.016 ± 0.001 | 0.030 ± 0.003 | 0.018 ± 0.002 | NS | < 0.05* | NS | < 0.05* | NS | < 0.05* |
| 27:0 | N/D | traces | traces | traces | NS | NS | NS | NS | NS | NS |
| 29:0 | N/D | traces | traces | traces | NS | NS | NS | NS | NS | NS |
| **Branched chain FAs** |  |  |  |  |  |  |  |  |  |  |
| iso 12-M-13:0 | 0.012 ± 0.001 | 0.006 ± 0.001 | 0.016 ± 0.003 | 0.009 ± 0.001 | < 0.05* | NS | NS | < 0.05* | NS | NS |
| iso 13-M-14:0 | 0.022 ± 0.002 | 0.020 ± 0.002 | 0.033 ± 0.003 | 0.024 ± 0.002 | NS | NS | NS | < 0.05* | NS | NS |
| iso 14-M-15:0 | 0.056 ± 0.005 | 0.050 ± 0.004 | 0.071 ± 0.005 | 0.055 ± 0.005 | NS | NS | NS | 0.008 | NS | NS |
| iso 15-M-16:0 | 0.093 ± 0.006 | 0.099 ± 0.007 | 0.121 ± 0.008 | 0.117 ± 0.010 | NS | NS | NS | NS | NS | NS |
| iso 21-M-21:0 | 0.006 ± 0.001 | 0.005 ± 0.001 | 0.008 ± 0.001 | 0.007 ± 0.001 | NS | NS | NS | NS | NS | NS |
| anteiso 12-M-14:0 | 0.036 ± 0.004 | 0.028 ± 0.002 | 0.068 ± 0.007 | 0.036 ± 0.003 | NS | < 0.05* | NS | < 0.05* | NS | < 0.05* |
| anteiso 14-M-16:0 | 0.063 ± 0.007 | 0.091 ± 0.007 | 0.126 ± 0.009 | 0.105 ± 0.009 | NS | < 0.001 | 0.005 | 0.014 | NS | NS |
| anteiso 16-M-18:0 | 0.033 ± 0.001 | 0.029 ± 0.002 | 0.036 ± 0.002 | 0.031 ± 0.002 | NS | NS | NS | NS | NS | NS |
| anteiso 20-M-22:0 | 0.008 ± 0.001 | 0.009 ± 0.001 | 0.010 ± 0.001 | 0.011 ± 0.001 | NS | NS | NS | NS | NS | NS |
| 4,8,12-M-13:0 | 0.011 ± 0.001 | 0.010 ± 0.001 | 0.021 ± 0.002 | 0.011 ± 0.001 | NS | < 0.05* | NS | < 0.05* | NS | < 0.05* |
| **Monounsaturated FAs** |  |  |  |  |  |  |  |  |  |  |
| 14:1 | 0.065 ± 0.009 | 0.050 ± 0.005 | 0.090 ± 0.010 | 0.062 ± 0.006 | NS | NS | NS | < 0.05* | NS | NS |
| 16:1 | 3.04 ± 0.123 | 3.71 ± 0.147 | 4.14 ± 0.238 | 3.79 ± 0.231 | < 0.05* | < 0.05* | NS | NS | NS | NS |
| 17:1 | 0.104 ± 0.007 | 0.173 ± 0.012 | 0.236 ± 0.012 | 0.197 ± 0.011 | < 0.05* | < 0.05* | < 0.05* | < 0.05* | NS | NS |
| 18:1 | 25.0 ± 0.444 | 27.6 ± 0.532 | 26.5 ± 0.494 | 27.2 ± 0.544 | 0.002 | NS | 0.025 | NS | NS | NS |
| 19:1 | 0.013 ± 0.002 | 0.015 ± 0.001 | 0.014 ± 0.001 | 0.016 ± 0.001 | NS | NS | NS | NS | NS | NS |
| 20:1 | 0.108 ± 0.008 | 0.159 ± 0.010 | 0.174 ± 0.010 | 0.212 ± 0.014 | 0.003 | < 0.001 | < 0.001 | NS | 0.005 | NS |
| 22:1 | 0.011 ± 0.002 | 0.013 ± 0.001 | 0.014 ± 0.001 | 0.017 ± 0.002 | NS | NS | < 0.05* | NS | NS | NS |
| 24:1 | 0.248 ± 0.017 | 0.279 ± 0.020 | 0.307 ± 0.016 | 0.316 ± 0.017 | NS | NS | NS | NS | NS | NS |
| **Polyunsaturated FAs** |  |  |  |  |  |  |  |  |  |  |
| 16:2 n-6 | 0.009 ± 0.001 | 0.009 ± 0.001 | 0.009 ± 0.000 | 0.009 ± 0.001 | NS | NS | NS | NS | NS | NS |
| 18:2 n-6 LA | 28.2 ± 0.627 | 24.2 ± 0.604 | 22.3 ± 0.657 | 22.3 ± 0.694 | < 0.001 | < 0.001 | < 0.001 | NS | NS | NS |
| 20:4 n-6 ARA | 5.83 ± 0.246 | 5.79 ± 0.204 | 5.95 ± 0.273 | 6.07 ± 0.322 | NS | NS | NS | NS | NS | NS |
| 20:3 n-6 DGLA | 1.18 ± 0.058 | 1.26 ± 0.070 | 1.48 ± 0.053 | 1.55 ± 0.061 | NS | 0.004 | < 0.001 | NS | 0.013 | NS |
| 20:2 n-6 | 0.109 ± 0.006 | 0.156 ± 0.011 | 0.200 ± 0.007 | 0.189 ± 0.005 | < 0.05* | < 0.05* | < 0.05* | < 0.05* | NS | NS |
| 22:4 n-6 AdA | 0.098 ± 0.005 | 0.107 ± 0.006 | 0.134 ± 0.006 | 0.139 ± 0.011 | NS | < 0.05* | < 0.05* | < 0.05* | NS | NS |
| 18:3 n-3 ALA | 0.248 ± 0.019 | 0.329 ± 0.026 | 0.367 ± 0.024 | 0.351 ± 0.035 | NS | < 0.05* | NS | NS | NS | NS |
| 20:5 n-3 EPA | 0.643 ± 0.055 | 0.878 ± 0.064 | 0.895 ± 0.054 | 0.894 ± 0.058 | 0.023 | 0.012 | 0.033 | NS | NS | NS |
| 20:4 n-3 ETA | 0.058 ± 0.002 | 0.061 ± 0.006 | 0.088 ± 0.006 | 0.101 ± 0.009 | NS | < 0.05* | < 0.05* | < 0.05* | < 0.05* | NS |
| 22:6 n-3 DHA | 1.19 ± 0.099 | 1.51 ± 0.095 | 1.56 ± 0.077 | 1.84 ± 0.088 | NS | 0.020 | < 0.001 | NS | NS | NS |
| 22:5 n-3 DPA | 0.290 ± 0.019 | 0.425 ± 0.023 | 0.393 ± 0.017 | 0.388 ± 0.014 | < 0.05* | < 0.05* | < 0.05* | NS | NS | NS |
| **Indices/groups** |  |  |  |  |  |  |  |  |  |  |
| Total SFAs | 32.0 ± 0.290 | 32.1 ± 0.265 | 34.9 ± 0.401 | 33.6 ± 0.407 | NS | < 0.05* | NS | < 0.05* | NS | NS |
| PUFA n-6/n-3 ratio | 15.8 ± 1.29 | 11.0 ± 0.543 | 9.19 ± 0.418 | 8.51 ± 0.369 | < 0.05* | < 0.05* | < 0.05* | NS | NS | NS |
| LA/18:1 ratio | 1.15 ± 0.035 | 0.878 ± 0.048 | 0.853 ± 0.036 | 0.832 ± 0.037 | < 0.05* | < 0.05* | < 0.05* | NS | NS | NS |
| 18:1/18:0 (SCD-1 index) | 3.49 ± 0.096 | 3.90 ± 0.115 | 3.36 ± 0.092 | 3.47 ± 0.107 | 0.027 | NS | NS | 0.001 | 0.030 | NS |
| 18:0/16:0 (ELOVL6 index) | 0.315 ± 0.007 | 0.312 ± 0.008 | 0.347 ± 0.008 | 0.351 ± 0.011 | NS | 0.033 | 0.028 | 0.012 | 0.011 | NS |
| ARA/DGLA (D5D index) | 5.147 ± 0.412 | 4.921 ± 0.306 | 4.164 ± 0.199 | 4.121 ± 0.260 | NS | NS | NS | NS | NS | NS |
| DGLA/LA (D6D index) | 0.042 ± 0.002 | 0.051 ± 0.004 | 0.068 ± 0.003 | 0.070 ± 0.003 | NS | < 0.001 | < 0.001 | 0.002 | 0.002 | NS |

Mean ± SEM, content [%], N/D: not detected; p-value from All Pairwise Multiple Comparison Procedures (Tukey Test); * p-value from Kruskal-Wallis One Way Analysis of Variance on Ranks followed by All Pairwise Multiple Comparison Procedures (Dunn's Method); NS: not significant; AdA - adrenic acid; ALA: α-linolenic acid; ARA: arachidonic acid; DGLA: dihomo-γ-linolenic acid; DHA: docosahexaenoic acid; D5D: delta-5-desaturase; D6D: delta-6-desaturase; DPA: docosapentaenoic acid; ELOVL6: fatty acid elongase 6; EPA: eicosapentaenoic acid; ETA: eicosatetraenoic acid; LA: linoleic acid; PUFA: polyunsaturated fatty acids; SCD-1: stearoyl-CoA desaturase-1; SFA: saturated fatty acids.

**Table S3. Comparison of serum fatty acid profiles between the age-matched control group and breast cancer patients preoperative and in follow-ups.**

|  | **Serum content [%]** | | | | **p-value for comparison** | | | | | |
| --- | --- | --- | --- | --- | --- | --- | --- | --- | --- | --- |
| **Fatty acid** | **Control**  **n = 18** | **Preoperative**  **n = 28** | **12M follow-up**  **n = 27** | **24M follow-up**  **n = 19** | **C vs Pre** | **C vs 12M** | **C vs 24M** | **Pre vs 12M** | **Pre vs 24M** | **12M vs 24M** |
| **Age** | 49.83 ± 1.84 | 55.54 ± 2.17 ( p = 0.051) | | |  | | |  | | |
| **Even chain FAs** |  |  |  |  |  |  |  |  |  |  |
| 8:0 | ND | traces | traces | traces | NS | NS | NS | NS | NS | NS |
| 10:0 | 0.016 ± 0.005 | 0.007 ± 0.001 | 0.004 ± 0.001 | 0.005 ± 0.001 | NS | < 0.05* | NS | NS | NS | NS |
| 12:0 | 0.100 ± 0.015 | 0.114 ± 0.017 | 0.215 ± 0.013 | 0.136 ± 0.016 | NS | < 0.05* | NS | < 0.05* | NS | < 0.05* |
| 14:0 | 1.10 ± 0.092 | 0.989 ± 0.051 | 1.69 ± 0.089 | 1.34 ± 0.061 | NS | < 0.05* | NS | < 0.05* | < 0.05* | NS |
| 16:0 | 22.9 ± 0.369 | 22.8 ± 0.216 | 22.9 ± 0.298 | 22.7 ± 0.368 | NS | NS | NS | NS | NS | NS |
| 18:0 | 7.30 ± 0.156 | 7.10 ± 0.133 | 7.90 ± 0.127 | 7.90 ± 0.158 | NS | 0.020 | 0.033 | < 0.001 | < 0.001 | NS |
| 20:0 | 0.076 ± 0.005 | 0.116 ± 0.010 | 0.167 ± 0.005 | 0.164 ± 0.006 | NS | < 0.05* | < 0.05* | < 0.05* | < 0.05* | NS |
| 22:0 | 0.173 ± 0.011 | 0.189 ± 0.013 | 0.250 ± 0.009 | 0.235 ± 0.007 | NS | < 0.05* | < 0.05* | < 0.05* | < 0.05* | NS |
| 24:0 | 0.136 ± 0.012 | 0.176 ± 0.015 | 0.266 ± 0.012 | 0.224 ± 0.006 | NS | < 0.05* | < 0.05* | < 0.05* | NS | NS |
| 26:0 | 0.012 ± 0.002 | 0.013 ± 0.001 | 0.031 ± 0.003 | 0.017 ± 0.001 | NS | < 0.05* | NS | < 0.05* | NS | < 0.05* |
| 28:0 | traces | traces | 0.013 ± 0.002 | 0.009 ± 0.001 | NS | NS | NS | NS | NS | NS |
| 30:0 | traces | traces | traces | traces | NS | NS | NS | NS | NS | NS |
| **Odd chain FAs** |  |  |  |  |  |  |  |  |  |  |
| 9:0 | ND | 0.012 ± 0.009 | traces | traces | NS | NS | NS | NS | NS | NS |
| 11:0 | 0.006 ± 0.001 | 0.003 ± 0.000 | 0.003 ± 0.000 | 0.003 ± 0.000 | NS | NS | NS | NS | NS | NS |
| 13:0 | 0.013 ± 0.002 | 0.010 ± 0.001 | 0.011 ± 0.001 | 0.010 ± 0.001 | NS | NS | NS | NS | NS | NS |
| 15:0 | 0.260 ± 0.017 | 0.270 ± 0.012 | 0.388 ± 0.020 | 0.296 ± 0.014 | NS | < 0.05* | NS | < 0.05* | NS | < 0.05* |
| 17:0 | 0.249 ± 0.011 | 0.289 ± 0.013 | 0.353 ± 0.010 | 0.329 ± 0.006 | NS | < 0.05* | < 0.05* | < 0.05* | NS | NS |
| 19:0 | 0.017 ± 0.002 | 0.018 ± 0.001 | 0.032 ± 0.002 | 0.026 ± 0.002 | NS | < 0.05* | < 0.05* | < 0.05* | < 0.05* | NS |
| 21:0 | 0.016 ± 0.002 | 0.016 ± 0.002 | 0.027 ± 0.002 | 0.029 ± 0.002 | NS | < 0.05* | < 0.05* | < 0.05* | < 0.05* | NS |
| 23:0 | 0.051 ± 0.005 | 0.073 ± 0.008 | 0.112 ± 0.005 | 0.099 ± 0.006 | NS | < 0.05* | < 0.05* | < 0.05* | NS | NS |
| 25:0 | 0.015 ± 0.002 | 0.016 ± 0.001 | 0.030 ± 0.003 | 0.018 ± 0.002 | NS | < 0.05* | NS | < 0.05* | NS | < 0.05* |
| 27:0 | ND | traces | traces | traces | NS | NS | NS | NS | NS | NS |
| 29:0 | ND | traces | traces | traces | NS | NS | NS | NS | NS | NS |
| **Branched chain FAs** |  |  |  |  |  |  |  |  |  |  |
| iso 12-M-13:0 | 0.012 ± 0.002 | 0.006 ± 0.001 | 0.016 ± 0.003 | 0.009 ± 0.001 | < 0.05* | NS | NS | < 0.05* | NS | NS |
| iso 13-M-14:0 | 0.022 ± 0.003 | 0.020 ± 0.002 | 0.033 ± 0.003 | 0.024 ± 0.002 | NS | NS | NS | < 0.05* | NS | NS |
| iso 14-M-15:0 | 0.058 ± 0.006 | 0.050 ± 0.004 | 0.071 ± 0.005 | 0.055 ± 0.005 | NS | NS | NS | 0.004 | NS | NS |
| iso 15-M-16:0 | 0.086 ± 0.008 | 0.099 ± 0.007 | 0.121 ± 0.008 | 0.117 ± 0.010 | NS | 0.026 | NS | NS | NS | NS |
| iso 21-M-21:0 | 0.005 ± 0.001 | 0.005 ± 0.001 | 0.008 ± 0.001 | 0.007 ± 0.001 | NS | NS | NS | NS | NS | NS |
| anteiso 12-M-14:0 | 0.035 ± 0.004 | 0.028 ± 0.002 | 0.068 ± 0.007 | 0.036 ± 0.003 | NS | < 0.05* | NS | < 0.05* | NS | < 0.05* |
| anteiso 14-M-16:0 | 0.063 ± 0.009 | 0.091 ± 0.007 | 0.126 ± 0.009 | 0.105 ± 0.009 | NS | < 0.001 | 0.011 | 0.012 | NS | NS |
| anteiso 16-M-18:0 | 0.033 ± 0.002 | 0.029 ± 0.002 | 0.036 ± 0.002 | 0.031 ± 0.002 | NS | NS | NS | NS | NS | NS |
| anteiso 20-M-22:0 | 0.007 ± 0.001 | 0.009 ± 0.001 | 0.010 ± 0.001 | 0.011 ± 0.001 | NS | NS | < 0.05* | NS | NS | NS |
| 4,8,12-M-13:0 | 0.011 ± 0.001 | 0.010 ± 0.001 | 0.021 ± 0.002 | 0.011 ± 0.001 | NS | < 0.05* | NS | < 0.05* | NS | < 0.05* |
| **Monounsaturated FAs** |  |  |  |  |  |  |  |  |  |  |
| 14:1 | 0.069 ± 0.011 | 0.050 ± 0.005 | 0.090 ± 0.010 | 0.062 ± 0.006 | NS | NS | NS | < 0.05* | NS | NS |
| 16:1 | 3.08 ± 0.137 | 3.71 ± 0.147 | 4.14 ± 0.238 | 3.79 ± 0.231 | NS | < 0.05* | NS | NS | NS | NS |
| 17:1 | 0.101 ± 0.008 | 0.173 ± 0.012 | 0.236 ± 0.012 | 0.197 ± 0.011 | < 0.001 | < 0.001 | < 0.001 | < 0.001 | NS | NS |
| 18:1 | 25.0 ± 0.591 | 27.6 ± 0.532 | 26.5 ± 0.494 | 27.2 ± 0.544 | 0.001 | NS | 0.049 | NS | NS | NS |
| 19:1 | 0.013 ± 0.002 | 0.015 ± 0.001 | 0.014 ± 0.001 | 0.016 ± 0.001 | NS | NS | NS | NS | NS | NS |
| 20:1 | 0.097 ± 0.008 | 0.159 ± 0.010 | 0.174 ± 0.010 | 0.212 ± 0.014 | < 0.001 | < 0.001 | < 0.001 | NS | 0.002 | NS |
| 22:1 | 0.012 ± 0.002 | 0.013 ± 0.001 | 0.014 ± 0.001 | 0.017 ± 0.002 | NS | NS | NS | NS | NS | NS |
| 24:1 | 0.236 ± 0.021 | 0.279 ± 0.020 | 0.307 ± 0.016 | 0.316 ± 0.017 | NS | NS | < 0.05* | NS | NS | NS |
| **Polyunsaturated FAs** |  |  |  |  |  |  |  |  |  |  |
| 16:2 n-6 | 0.009 ± 0.001 | 0.009 ± 0.001 | 0.009 ± 0.000 | 0.009 ± 0.001 | NS | NS | NS | NS | NS | NS |
| 18:2 n-6 LA | 28.4 ± 0.772 | 24.2 ± 0.604 | 22.3 ± 0.657 | 22.3 ± 0.694 | < 0.001 | < 0.001 | < 0.001 | NS | NS | NS |
| 20:4 n-6 ARA | 5.78 ± 0.254 | 5.79 ± 0.204 | 5.95 ± 0.273 | 6.07 ± 0.322 | NS | NS | NS | NS | NS | NS |
| 20:3 n-6 DGLA | 1.14 ± 0.060 | 1.26 ± 0.070 | 1.48 ± 0.053 | 1.55 ± 0.061 | NS | 0.001 | < 0.001 | NS | 0.015 | NS |
| 20:2 n-6 | 0.107 ± 0.007 | 0.156 ± 0.011 | 0.200 ± 0.007 | 0.189 ± 0.005 | < 0.05* | < 0.05* | < 0.05* | < 0.05* | NS | NS |
| 22:4 n-6 AdA | 0.098 ± 0.007 | 0.107 ± 0.006 | 0.134 ± 0.006 | 0.139 ± 0.011 | NS | 0.009 | 0.005 | 0.020 | 0.010 | NS |
| 18:3 n-3 ALA | 0.257 ± 0.024 | 0.329 ± 0.026 | 0.367 ± 0.024 | 0.351 ± 0.035 | NS | 0.034 | NS | NS | NS | NS |
| 20:5 n-3 EPA | 0.657 ± 0.062 | 0.878 ± 0.064 | 0.895 ± 0.054 | 0.894 ± 0.058 | NS | 0.012 | NS | NS | NS | NS |
| 20:4 n-3 ETA | 0.058 ± 0.003 | 0.061 ± 0.006 | 0.088 ± 0.006 | 0.101 ± 0.009 | NS | < 0.05* | < 0.05* | < 0.05* | < 0.05* | NS |
| 22:6 n-3 DHA | 1.11 ± 0.097 | 1.51 ± 0.095 | 1.56 ± 0.077 | 1.84 ± 0.088 | 0.005 | 0.004 | < 0.001 | NS | NS | NS |
| 22:5 n-3 DPA | 0.288 ± 0.022 | 0.425 ± 0.023 | 0.393 ± 0.017 | 0.388 ± 0.014 | < 0.001 | 0.003 | 0.013 | NS | NS | NS |
| **Indices/groups** |  |  |  |  |  |  |  |  |  |  |
| Total SFAs | 32.0 ± 0.373 | 32.1 ± 0.265 | 34.9 ± 0.401 | 33.6 ± 0.407 | NS | < 0.05* | NS | < 0.05* | NS | NS |
| PUFA n-6/n-3 ratio | 14.32 ± 0.83 | 11.0 ± 0.543 | 9.19 ± 0.418 | 8.51 ± 0.369 | 0.001 | < 0.001 | < 0.001 | 0.037 | 0.017 | NS |
| LA/18:1 ratio | 1.17 ± 0.046 | 0.878 ± 0.048 | 0.853 ± 0.036 | 0.832 ± 0.037 | < 0.05* | < 0.05* | < 0.05* | NS | NS | NS |
| 18:1/18:0 (SCD-1 index) | 3.43 ± 0.116 | 3.90 ± 0.115 | 3.36 ± 0.092 | 3.47 ± 0.107 | 0.020 | NS | NS | 0.002 | 0.033 | NS |
| 18:0/16:0 (ELOVL6 index) | 0.315 ± 0.007 | 0.312 ± 0.008 | 0.347 ± 0.008 | 0.351 ± 0.011 | NS | NS | NS | 0.013 | 0.012 | NS |
| ARA/DGLA (D5D index) | 5.104 ± 0.368 | 4.921 ± 0.306 | 4.164 ± 0.199 | 4.121 ± 0.260 | NS | NS | NS | NS | NS | NS |
| DGLA/LA (D6D index) | 0.041 ± 0.002 | 0.051 ± 0.004 | 0.068 ± 0.003 | 0.070 ± 0.003 | NS | < 0.001 | < 0.001 | 0.002 | 0.002 | NS |

Mean ± SEM, content [%], N/D: not detected; p-value from All Pairwise Multiple Comparison Procedures (Tukey Test); * p-value from Kruskal-Wallis One Way Analysis of Variance on Ranks followed by All Pairwise Multiple Comparison Procedures (Dunn's Method); NS: not significant; AdA - adrenic acid; ALA: α-linolenic acid; ARA: arachidonic acid; DGLA: dihomo-γ-linolenic acid; DHA: docosahexaenoic acid; D5D: delta-5-desaturase; D6D: delta-6-desaturase; DPA: docosapentaenoic acid; ELOVL6: fatty acid elongase 6; EPA: eicosapentaenoic acid; ETA: eicosatetraenoic acid; LA: linoleic acid; PUFA: polyunsaturated fatty acids; SCD-1: stearoyl-CoA desaturase-1; SFA: saturated fatty acids.

**Table S4. Comparison of fatty acid serum content in paired samples from preoperative patients and 12 months follow-up.**

| **Fatty acid** | **Preoperative**  **n = 27** | **12M follow-up**  **n = 27** | **p-value** |
| --- | --- | --- | --- |
| 8:0 | traces | traces | - |
| 10:0 | 0.007 ± 0.001 | 0.004 ± 0.001 | 0.278* |
| 12:0 | 0.118 ± 0.017 | 0.215 ± 0.013 | < 0.001 |
| 14:0 | 0.997 ± 0.052 | 1.69 ± 0.089 | < 0.001 |
| 16:0 | 22.9 ± 0.223 | 22.9 ± 0.298 | 0.861 |
| 18:0 | 7.06 ± 0.133 | 7.90 ± 0.127 | < 0.001 |
| 20:0 | 0.113 ± 0.010 | 0.167 ± 0.005 | < 0.001 |
| 22:0 | 0.187 ± 0.014 | 0.250 ± 0.009 | < 0.001 |
| 24:0 | 0.174 ± 0.016 | 0.266 ± 0.012 | < 0.001 |
| 26:0 | 0.012 ± 0.001 | 0.031 ± 0.003 | < 0.001 |
| 28:0 | traces | 0.013 ± 0.002 | 0.063* |
| 30:0 | traces | traces | - |
| **ECFA** | 31.3 ± 0.230 | 33.5 ± 0.380 | < 0.001 |
| 9:0 | traces | traces | - |
| 11:0 | 0.003 ± 0.000 | 0.003 ± 0.000 | 1.000* |
| 13:0 | 0.011 ± 0.001 | 0.011 ± 0.001 | 0.922* |
| 15:0 | 0.270 ± 0.012 | 0.388 ± 0.020 | < 0.001 |
| 17:0 | 0.288 ± 0.013 | 0.353 ± 0.010 | < 0.001* |
| 19:0 | 0.018 ± 0.001 | 0.032 ± 0.002 | < 0.001* |
| 21:0 | 0.016 ± 0.002 | 0.027 ± 0.002 | < 0.001* |
| 23:0 | 0.070 ± 0.008 | 0.112 ± 0.005 | < 0.001 |
| 25:0 | 0.015 ± 0.001 | 0.030 ± 0.003 | < 0.001 |
| 27:0 | traces | traces | - |
| 29:0 | traces | traces | - |
| **OCFA** | 0.668 ± 0.032 | 0.953 ± 0.034 | < 0.001 |
| iso 12-M-13:0 | 0.006 ± 0.001 | 0.016 ± 0.003 | < 0.001* |
| iso 13-M-14:0 | 0.021 ± 0.002 | 0.033 ± 0.003 | 0.004 |
| iso 14-M-15:0 | 0.050 ± 0.004 | 0.071 ± 0.005 | < 0.001 |
| iso 15-M-16:0 | 0.098 ± 0.007 | 0.121 ± 0.008 | 0.003 |
| iso 21-M-21:0 | 0.005 ± 0.001 | 0.008 ± 0.001 | 0.021* |
| **iso BCFA** | 0.176 ± 0.013 | 0.248 ± 0.017 | < 0.001 |
| anteiso 12-M-14:0 | 0.028 ± 0.002 | 0.068 ± 0.007 | < 0.001* |
| anteiso 14-M-16:0 | 0.092 ± 0.008 | 0.126 ± 0.009 | 0.002 |
| anteiso 16-M-18:0 | 0.029 ± 0.002 | 0.036 ± 0.002 | 0.034 |
| anteiso 20-M-22:0 | 0.009 ± 0.001 | 0.010 ± 0.001 | 0.380* |
| **anteiso BCFA** | 0.153 ± 0.011 | 0.238 ± 0.018 | < 0.001 |
| 4,8,12-M-13:0 | 0.010 ± 0.001 | 0.021 ± 0.002 | < 0.001* |
| **BCFA** | 0.333 ± 0.022 | 0.505 ± 0.036 | < 0.001 |
| **SFA** | 32.1 ± 0.265 | 34.9 ± 0.401 | < 0.001 |
| 14:1 | 0.051 ± 0.005 | 0.090 ± 0.010 | 0.003 |
| 16:1 | 3.73 ± 0.151 | 4.14 ± 0.238 | 0.067* |
| CPOA2H=17:1 | 0.173 ± 0.012 | 0.236 ± 0.012 | < 0.001 |
| 18:1 | 27.7 ± 0.546 | 26.5 ± 0.494 | 0.028 |
| 19:1 | 0.016 ± 0.001 | 0.014 ± 0.001 | 0.588* |
| 20:1 | 0.157 ± 0.010 | 0.174 ± 0.010 | 0.014 |
| 22:1 | 0.012 ± 0.001 | 0.014 ± 0.001 | 0.577* |
| 24:1 | 0.272 ± 0.019 | 0.307 ± 0.016 | 0.197 |
| **MUFA** | 32.1 ± 0.546 | 31.2 ± 0.588 | 0.154* |
| 16:2 n-6 | 0.009 ± 0.001 | 0.009 ± 0.000 | 0.820* |
| 18:2 n-6 LA | 24.2 ± 0.628 | 22.3 ± 0.657 | < 0.001 |
| 20:4 n-6 ARA | 5.76 ± 0.209 | 5.95 ± 0.273 | 0.478 |
| 20:3 n-6 DGLA | 1.25 ± 0.070 | 1.48 ± 0.053 | < 0.001 |
| 20:2 n-6 | 0.151 ± 0.011 | 0.200 ± 0.007 | < 0.001 |
| 22:4 n-6 AdA | 0.106 ± 0.006 | 0.134 ± 0.006 | < 0.001 |
| **n-6 PUFA** | 31.8 ± 0.670 | 30.0 ± 0.831 | 0.015 |
| 18:3 n-3 ALA | 0.332 ± 0.027 | 0.367 ± 0.024 | 0.069 |
| 20:5 n-3 EPA | 0.871 ± 0.067 | 0.895 ± 0.054 | 0.371 |
| 20:4 n-3 ETA | 0.060 ± 0.006 | 0.088 ± 0.006 | < 0.001 |
| 22:6 n-3 DHA | 1.47 ± 0.091 | 1.56 ± 0.077 | 0.293 |
| 22:5 n-3 DPA | 0.423 ± 0.024 | 0.393 ± 0.017 | 0.191 |
| **n-3 PUFA** | 3.02 ± 0.161 | 3.26 ± 0.126 | 0.292 |

Mean ± SEM, content [%], p value from paired two-tailed t-test for data with normal distribution, *p value from Wilcoxon Signed Rank Test. AdA: adrenic acid; ALA: α-linolenic acid; ARA: arachidonic acid; BCFA: branched chain fatty acids; ECFA: even chain saturated fatty acids; DGLA: dihomo-γ-linolenic acid; DHA: docosahexaenoic acid; DPA: docosapentaenoic acid; EPA: eicosapentaenoic acid; ETA: eicosatetraenoic acid; LA: linoleic acid; MUFA: monounsaturated fatty acids; OCFA: odd chain saturated fatty acids; PUFA: polyunsaturated fatty acids.

**Table S5. Comparison of fatty acid serum content in paired samples from preoperative patients and 24 months follow-up.**

| **Fatty acid** | **Preoperative**  **n = 19** | **24M follow-up**  **n = 19** | **p-value** |
| --- | --- | --- | --- |
| 8:0 | traces | traces | - |
| 10:0 | 0.008 ± 0.001 | 0.005 ± 0.001 | 0.500* |
| 12:0 | 0.111 ± 0.015 | 0.136 ± 0.016 | 0.360 |
| 14:0 | 0.975 ± 0.057 | 1.34 ± 0.061 | 0.002 |
| 16:0 | 22.8 ± 0.284 | 22.7 ± 0.368 | 0.799 |
| 18:0 | 7.16 ± 0.143 | 7.90 ± 0.158 | 0.003 |
| 20:0 | 0.125 ± 0.011 | 0.164 ± 0.006 | 0.009 |
| 22:0 | 0.197 ± 0.015 | 0.235 ± 0.007 | 0.052 |
| 24:0 | 0.190 ± 0.018 | 0.224 ± 0.006 | 0.200 |
| 26:0 | 0.014 ± 0.002 | 0.017 ± 0.001 | 0.188 |
| 28:0 | traces | 0.009 ± 0.001 | 0.363 |
| 30:0 | traces | traces | - |
| **ECFA** | 31.3 ± 0.300 | 32.6 ± 0.406 | 0.164 |
| 9:0 | traces | traces | - |
| 11:0 | 0.004 ± 0.001 | 0.003 ± 0.000 | 1.000* |
| 13:0 | 0.011 ± 0.001 | 0.010 ± 0.001 | 0.375* |
| 15:0 | 0.268 ± 0.015 | 0.296 ± 0.014 | 0.039 |
| 17:0 | 0.297 ± 0.015 | 0.329 ± 0.006 | 0.017 |
| 19:0 | 0.018 ± 0.002 | 0.026 ± 0.002 | 0.035 |
| 21:0 | 0.015 ± 0.002 | 0.029 ± 0.002 | < 0.001 |
| 23:0 | 0.076 ± 0.009 | 0.099 ± 0.006 | 0.052 |
| 25:0 | 0.016 ± 0.002 | 0.018 ± 0.002 | 0.524* |
| 27:0 | traces | traces | - |
| 29:0 | traces | traces | - |
| **OCFA** | **0.664 ± 0.035** | **0.816 ± 0.024** | **< 0.001** |
| iso 12-M-13:0 | 0.006 ± 0.001 | 0.009 ± 0.001 | 0.094* |
| iso 13-M-14:0 | 0.021 ± 0.003 | 0.024 ± 0.002 | 0.042 |
| iso 14-M-15:0 | 0.053 ± 0.005 | 0.055 ± 0.005 | 0.628 |
| iso 15-M-16:0 | 0.099 ± 0.007 | 0.117 ± 0.010 | 0.102 |
| iso 21-M-21:0 | 0.005 ± 0.001 | 0.007 ± 0.001 | 0.275* |
| **iso BCFA** | **0.184 ± 0.015** | **0.211 ± 0.017** | **0.153** |
| anteiso 12-M-14:0 | 0.027 ± 0.003 | 0.036 ± 0.003 | 0.001 |
| anteiso 14-M-16:0 | 0.095 ± 0.009 | 0.105 ± 0.009 | 0.313 |
| anteiso 16-M-18:0 | 0.030 ± 0.002 | 0.031 ± 0.002 | 0.848 |
| anteiso 20-M-22:0 | 0.009 ± 0.001 | 0.011 ± 0.001 | 0.219* |
| **anteiso BCFA** | **0.157 ± 0.013** | **0.178 ± 0.013** | **0.019** |
| 4,8,12-M-13:0 | 0.010 ± 0.001 | 0.011 ± 0.001 | 0.688* |
| **BCFA** | **0.343 ± 0.027** | **0.390 ± 0.030** | **0.022** |
| **SFA** | **32.0 ± 0.371** | **33.6 ± 0.408** | **0.041** |
| 14:1 | 0.049 ± 0.006 | 0.062 ± 0.006 | 0.093 |
| 16:1 | 3.70 ± 0.183 | 3.79 ± 0.231 | 0.050 |
| CPOA2H=17:1 | 0.176 ± 0.014 | 0.197 ± 0.011 | 0.159 |
| 18:1 | 27.3 ± 0.672 | 27.2 ± 0.544 | 0.809 |
| 19:1 | 0.014 ± 0.001 | 0.016 ± 0.001 | 0.206* |
| 20:1 | 0.156 ± 0.013 | 0.212 ± 0.014 | < 0.001 |
| 22:1 | 0.014 ± 0.002 | 0.017 ± 0.002 | 0.413* |
| 24:1 | 0.291 ± 0.024 | 0.316 ± 0.017 | 0.429 |
| **MUFA** | **31.7 ± 0.705** | **31.2 ± 0.597** | **0.529** |
| 16:2 n-6 | 0.008 ± 0.001 | 0.009 ± 0.001 | 0.469* |
| 18:2 n-6 LA | 24.3 ± 0.775 | 22.3 ± 0.694 | 0.007 |
| 20:4 n-6 ARA | 5.99 ± 0.240 | 6.07 ± 0.322 | 0.547 |
| 20:3 n-6 DGLA | 1.26 ± 0.081 | 1.55 ± 0.061 | < 0.001 |
| 20:2 n-6 | 0.156 ± 0.015 | 0.189 ± 0.005 | 0.105 |
| 22:4 n-6 AdA | 0.109 ± 0.008 | 0.139 ± 0.011 | 0.020 |
| **n-6 PUFA** | **32.2 ± 0.786** | **29.8 ± 1.10** | **0.057** |
| 18:3 n-3 ALA | 0.360 ± 0.030 | 0.351 ± 0.035 | 0.753 |
| 20:5 n-3 EPA | 0.939 ± 0.083 | 0.894 ± 0.058 | 0.719 |
| 20:4 n-3 ETA | 0.065 ± 0.007 | 0.101 ± 0.009 | 0.004 |
| 22:6 n-3 DHA | 1.58 ± 0.122 | 1.84 ± 0.088 | 0.019 |
| 22:5 n-3 DPA | 0.443 ± 0.029 | 0.388 ± 0.014 | 0.153 |
| **n-3 PUFA** | **3.28 ± 0.203** | **3.47 ± 0.114** | **0.147** |

Mean ± SEM, content [%], p value from paired two-tailed t-test for data with normal distribution, *p value from Wilcoxon Signed Rank Test. AdA: adrenic acid; ALA: α-linolenic acid; ARA: arachidonic acid; BCFA: branched chain fatty acids; ECFA: even chain saturated fatty acids; DGLA: dihomo-γ-linolenic acid; DHA: docosahexaenoic acid; DPA: docosapentaenoic acid; EPA: eicosapentaenoic acid; ETA: eicosatetraenoic acid; LA: linoleic acid; MUFA: monounsaturated fatty acids; OCFA: odd chain saturated fatty acids; PUFA: polyunsaturated fatty acids.

**Table S6. Comparison of fatty acid serum content in paired samples from patients in 12 months and 24 months follow-up.**

| **Fatty acid** | **12M follow-up**  **n = 18** | **24M follow-up**  **n = 18** | **p-value** |
| --- | --- | --- | --- |
| 8:0 | traces | traces | - |
| 10:0 | 0.005 ± 0.001 | 0.004 ± 0.001 | 0.500* |
| 12:0 | 0.218 ± 0.016 | 0.126 ± 0.013 | < 0.001 |
| 14:0 | 1.71 ± 0.102 | 1.33 ± 0.063 | < 0.001 |
| 16:0 | 23.1 ± 0.364 | 22.8 ± 0.366 | 0.514 |
| 18:0 | 7.77 ± 0.152 | 7.86 ± 0.159 | 0.655 |
| 20:0 | 0.169 ± 0.006 | 0.161 ± 0.005 | 0.293 |
| 22:0 | 0.256 ± 0.010 | 0.233 ± 0.007 | 0.001 |
| 24:0 | 0.274 ± 0.015 | 0.223 ± 0.006 | < 0.001 |
| 26:0 | 0.029 ± 0.003 | 0.018 ± 0.002 | < 0.001 |
| 28:0 | 0.011 ± 0.001 | 0.009 ± 0.001 | 0.094* |
| 30:0 | traces | traces | - |
| **ECFA** | **33.5 ± 0.423** | **32.6 ± 0.434** | **0.170** |
| 9:0 | traces | traces | - |
| 11:0 | 0.003 ± 0.000 | 0.003 ± 0.000 | 1.00* |
| 13:0 | 0.011 ± 0.001 | 0.010 ± 0.001 | 0.625 |
| 15:0 | 0.391 ± 0.026 | 0.294 ± 0.015 | < 0.001 |
| 17:0 | 0.358 ± 0.013 | 0.329 ± 0.007 | 0.004 |
| 19:0 | 0.032 ± 0.002 | 0.026 ± 0.002 | 0.021* |
| 21:0 | 0.028 ± 0.002 | 0.029 ± 0.002 | 0.322* |
| 23:0 | 0.116 ± 0.005 | 0.099 ± 0.006 | 0.006 |
| 25:0 | 0.031 ± 0.003 | 0.018 ± 0.002 | 0.002 |
| 27:0 | traces | traces | - |
| 29:0 | traces | traces | - |
| **OCFA** | **0.964 ± 0.041** | **0.813 ± 0.025** | **< 0.001*** |
| iso 12-M-13:0 | 0.014 ± 0.002 | 0.008 ± 0.001 | 0.010* |
| iso 13-M-14:0 | 0.032 ± 0.003 | 0.024 ± 0.002 | 0.051 |
| iso 14-M-15:0 | 0.068 ± 0.006 | 0.055 ± 0.005 | 0.008* |
| iso 15-M-16:0 | 0.120 ± 0.010 | 0.114 ± 0.010 | 0.518 |
| iso 21-M-21:0 | 0.007 ± 0.001 | 0.007 ± 0.001 | 0.750* |
| **iso BCFA** | **0.241 ± 0.020** | **0.209 ± 0.017** | **0.055*** |
| anteiso 12-M-14:0 | 0.065 ± 0.008 | 0.034 ± 0.003 | < 0.001* |
| anteiso 14-M-16:0 | 0.125 ± 0.011 | 0.105 ± 0.009 | 0.016 |
| anteiso 16-M-18:0 | 0.036 ± 0.003 | 0.031 ± 0.002 | 0.131* |
| anteiso 20-M-22:0 | 0.010 ± 0.001 | 0.011 ± 0.001 | 0.438* |
| **anteiso BCFA** | **0.232 ± 0.021** | **0.176 ± 0.014** | **0.004** |
| 4,8,12-M-13:0 | 0.021 ± 0.002 | 0.011 ± 0.001 | 0.001 |
| **BCFA** | **0.490 ± 0.042** | **0.385 ± 0.031** | **0.006** |
| **SFA** | **34.9 ± 0.431** | **33.6 ± 0.452** | **0.185** |
| 14:1 | 0.096 ± 0.012 | 0.061 ± 0.006 | 0.004* |
| 16:1 | 4.18 ± 0.286 | 3.87 ± 0.232 | 0.132 |
| CPOA2H=17:1 | 0.232 ± 0.015 | 0.201 ± 0.011 | 0.008* |
| 18:1 | 26.2 ± 0.600 | 27.3 ± 0.567 | 0.067* |
| 19:1 | 0.014 ± 0.001 | 0.017 ± 0.001 | 0.275 |
| 20:1 | 0.169 ± 0.011 | 0.208 ± 0.014 | 0.006 |
| 22:1 | 0.014 ± 0.001 | 0.017 ± 0.002 | 0.218 |
| 24:1 | 0.310 ± 0.019 | 0.313 ± 0.018 | 0.870 |
| **MUFA** | **31.1 ± 0.811** | **31.4 ± 0.617** | **0.502** |
| 16:2 n-6 | 0.009 ± 0.001 | 0.009 ± 0.001 | 0.875* |
| 18:2 n-6 LA | 22.4 ± 0.799 | 22.0 ± 0.677 | 0.530 |
| 20:4 n-6 ARA | 6.06 ± 0.368 | 6.07 ± 0.342 | 0.434 |
| 20:3 n-6 DGLA | 1.50 ± 0.059 | 1.57 ± 0.062 | 0.991 |
| 20:2 n-6 | 0.203 ± 0.008 | 0.189 ± 0.006 | 0.801 |
| 22:4 n-6 AdA | 0.135 ± 0.008 | 0.143 ± 0.012 | 0.808 |
| **n-6 PUFA** | **30.3 ± 1.062** | **29.4 ± 1.107** | **0.022** |
| 18:3 n-3 ALA | 0.380 ± 0.030 | 0.361 ± 0.035 | 0.134 |
| 20:5 n-3 EPA | 0.884 ± 0.067 | 0.898 ± 0.062 | 0.865 |
| 20:4 n-3 ETA | 0.089 ± 0.007 | 0.103 ± 0.009 | 0.005* |
| 22:6 n-3 DHA | 1.65 ± 0.088 | 1.83 ± 0.093 | 0.056 |
| 22:5 n-3 DPA | 0.390 ± 0.020 | 0.391 ± 0.015 | 0.673 |
| **n-3 PUFA** | **3.38 ± 0.157** | **3.48 ± 0.122** | **0.612** |

Mean ± SEM, content [%], p value from paired two-tailed t-test for data with normal distribution, *p value from Wilcoxon Signed Rank Test. AdA: adrenic acid; ALA: α-linolenic acid; ARA: arachidonic acid; BCFA: branched chain fatty acids; ECFA: even chain saturated fatty acids; DGLA: dihomo-γ-linolenic acid; DHA: docosahexaenoic acid; DPA: docosapentaenoic acid; EPA: eicosapentaenoic acid; ETA: eicosatetraenoic acid; LA: linoleic acid; MUFA: monounsaturated fatty acids; OCFA: odd chain saturated fatty acids; PUFA: polyunsaturated fatty acids.

**Table S7. Discriminant analysis models summary.**

| **Comparison** | **N** | **PCs/LVs** | **R^2^X(cum)** | **R^2^Y(cum)** | **Q^2^(cum)** | **CV-ANOVA p-value** |
| --- | --- | --- | --- | --- | --- | --- |
| **Preoperative vs 12M follow-up** | 54 | 2 | 0.844 | 0.463 | 0.389 | 2.2 ⨯ 10^-4^ |
| **Preoperative vs 24M follow-up** | 38 | 2 | 0.729 | 0.457 | 0.367 | 3.9 ⨯ 10^-3^ |
| **12M follow-up vs 24M follow-up** | 36 | 2 | 0.902 | 0.393 | 0.089 | 0.900 |
| **Control vs preoperative** | 53 | 2 | 0.815 | 0.504 | 0.411 | 3.3 ⨯ 10^-5^ |
| **Control vs 12M follow-up** | 52 | 3 | 0.821 | 0.862 | 0.815 | 6.3 ⨯ 10^-15^ |
| **Control vs 24M follow-up** | 44 | 3 | 0.805 | 0.903 | 0.847 | 6.5 ⨯ 10^-14^ |

CV-ANOVA: cross-validated one way analysis of variance; LV: latent variable; PC: principal component; PLS-DA: partial least squares discriminant analysis.

**Table S8. P-values from two-way t-Student’s test for comparisons between breast cancer patients’ serum fatty acids levels at different time points versus the control group.**

| **Fatty acid** | **vs preoperative patients**  **n = 28** | **vs 12M follow-up**  **n = 27** | **vs 24M follow-up**  **n = 19** |
| --- | --- | --- | --- |
| 10:0 | 0.058* | < 0.001* | < 0.001* |
| 12:0 | 0.208* | < 0.001 | 0.012* |
| 14:0 | 0.725 | < 0.001 | 0.003 |
| 16:0 | 0.914 | 0.901 | 0.732 |
| 18:0 | 0.602 | < 0.001 | 0.002 |
| 20:0 | <0.001 | < 0.001 | < 0.001 |
| 22:0 | 0.154 | < 0.001 | < 0.001 |
| 24:0 | 0.017 | < 0.001 | < 0.001 |
| 26:0 | 0.740 | < 0.001 | 0.022 |
| 28:0 | 0.481* | < 0.001* | 0.007 |
| **ECFA** | **0.864** | **< 0.001** | **0.016** |
| 11:0 | 0.007* | 0.022* | < 0.001* |
| 13:0 | 0.746* | 0.867* | 0.382* |
| 15:0 | 0.407 | < 0.001* | 0.055 |
| 17:0 | 0.009* | < 0.001 | < 0.001 |
| 19:0 | 0.183* | < 0.001* | < 0.001 |
| 21:0 | 0.489* | < 0.001* | < 0.001 |
| 23:0 | 0.010 | < 0.001* | < 0.001 |
| 25:0 | 0.663 | < 0.001 | 0.323* |
| **OCFA** | **0.098** | **< 0.001** | **< 0.001** |
| iso 12-M-13:0 | < 0.001* | 0.323* | 0.052* |
| iso 13-M-14:0 | 0.610 | 0.011* | 0.603 |
| iso 14-M-15:0 | 0.331 | 0.042 | 0.916 |
| iso 15-M-16:0 | 0.496 | 0.015 | 0.045 |
| iso 21-M-21:0 | 0.563* | 0.086* | 0.418* |
| **iso BCFA** | **0.836** | **0.002** | **0.140** |
| anteiso 12-M-14:0 | 0.092* | < 0.001* | 0.472* |
| anteiso 14-M-16:0 | 0.008 | < 0.001 | < 0.001 |
| anteiso 16-M-18:0 | 0.090 | 0.273 | 0.335* |
| anteiso 20-M-22:0 | 0.357* | 0.037* | 0.007* |
| **anteiso BCFA** | **0.195** | **< 0.001** | **0.010** |
| 4,8,12-M-13:0 | 0.364* | < 0.001* | 0.876 |
| **BCFA** | **0.555** | **< 0.001** | **0.041** |
| **SFA** | **0.754** | **< 0.001** | **0.005** |
| 14:1 | 0.132 | 0.059* | 0.565* |
| 16:1 | 0.005* | < 0.001* | 0.029* |
| CPOA2H=17:1 | < 0.001 | < 0.001* | < 0.001* |
| 18:1 | < 0.001 | 0.025 | 0.003 |
| 19:1 | 0.044* | 0.066* | 0.006* |
| 20:1 | < 0.001 | < 0.001 | < 0.001 |
| 22:1 | 0.279* | 0.140* | 0.003* |
| 24:1 | 0.149 | 0.013 | 0.008 |
| **MUFA** | **< 0.001*** | **0.001** | **0.001** |
| 16:2 n-6 | 0.812* | 0.620* | 0.710* |
| 18:2 n-6 LA | < 0.001 | < 0.001 | < 0.001 |
| 20:4 n-6 ARA | 0.839 | < 0.001 | 0.553 |
| 20:3 n-6 DGLA | 0.236 | < 0.001 | < 0.001 |
| 20:2 n-6 | 0.001 | < 0.001 | < 0.001 |
| 22:4 n-6 AdA | 0.335 | < 0.001 | 0.003 |
| **n-6 PUFA** | **< 0.001** | **< 0.001** | **< 0.001** |
| 18:3 n-3 ALA | 0.019 | < 0.001 | 0.015 |
| 20:5 n-3 EPA | 0.008 | 0.002 | 0.004 |
| 20:4 n-3 ETA | 0.595 | < 0.001 | < 0.001* |
| 22:6 n-3 DHA | 0.020 | 0.004 | < 0.001 |
| 22:5 n-3 DPA | < 0.001 | < 0.001 | < 0.001 |
| **n-3 PUFA** | **0.021** | **< 0.001** | **< 0.001** |

P value was calculated from two-tailed t-test for data with normal distribution, for groups with equal or unequal variance based on results from Fisher test, *p value from Mann-Whitney Rank Sum Test for non-parametric data. AdA: adrenic acid; ALA: α-linolenic acid; ARA: arachidonic acid; BCFA: branched chain fatty acids; ECFA: even chain saturated fatty acids; DGLA: dihomo-γ-linolenic acid; DHA: docosahexaenoic acid; DPA: docosapentaenoic acid; EPA: eicosapentaenoic acid; ETA: eicosatetraenoic acid; LA: linoleic acid; MUFA: monounsaturated fatty acids; OCFA: odd chain saturated fatty acids; PUFA: polyunsaturated fatty acids.


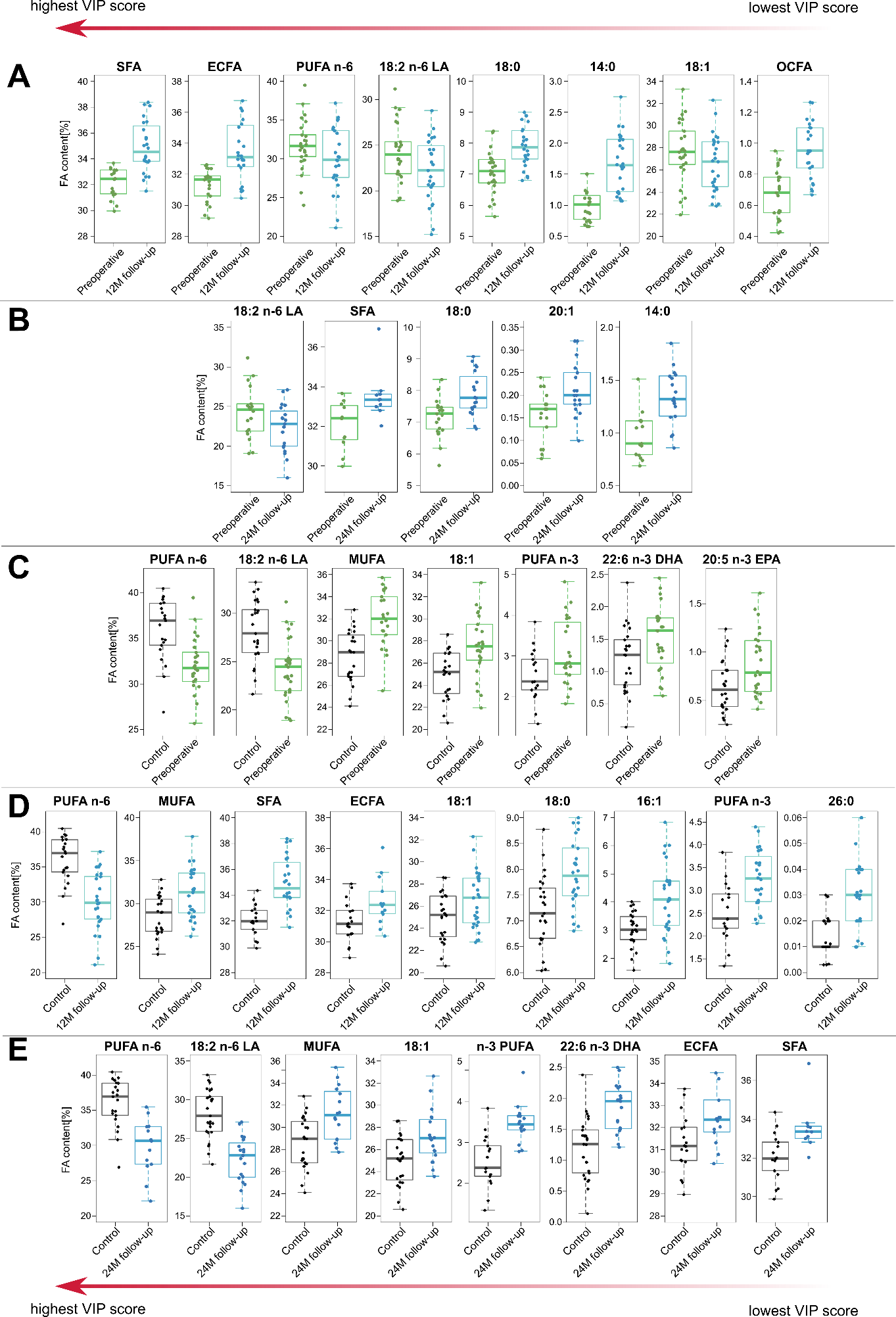


**Figure S1. Boxplots for variables with VIP scores above 1 in significant PLS-DA models.** Box lower edge: first quartile; box upper edge: third quartile; whiskers: 1.5 x interquartile range; bar: median. (A) patients before treatment and after 12 months (n = 27), (B) patients before treatment and after 24 months (n = 19), (C) preoperative patients (n = 28) and control (n = 25), (D) patients at 12M follow-up (n = 27) and controls (n = 25), (E) patients at 24M follow-up (n=19) and controls (n = 25).
